# Short tandem repeats, segmental duplications, gene deletion, and genomic instability in a rapidly diversified immune gene family

**DOI:** 10.1186/s12864-016-3241-x

**Published:** 2016-11-09

**Authors:** Matan Oren, Megan A. Barela Hudgell, Brian D’Allura, Jacob Agronin, Alexandra Gross, Daniele Podini, L. Courtney Smith

**Affiliations:** 1 The Department of Biological Sciences, George Washington University, Washington, DC USA; 2 Department of Forensic Sciences, George Washington University, Washington, DC USA; 3 Present Address: Pennsylvania College of Optometry, Salus University, Elkins Park, PA USA

**Keywords:** *Strongylocentrotus purpuratus*, Gene diversification, Allelic mispairing, Genomic instability *Sp185/333*, Illumina vs. PacBio

## Abstract

**Background:**

Genomic regions with repetitive sequences are considered unstable and prone to swift DNA diversification processes. A highly diverse immune gene family of the sea urchin (*Strongylocentrotus purpuratus*), called *Sp185/333*, is composed of clustered genes with similar sequence as well as several types of repeats ranging in size from short tandem repeats (STRs) to large segmental duplications. This repetitive structure may have been the basis for the incorrect assembly of this gene family in the sea urchin genome sequence. Consequently, we have resolved the structure of the family and profiled the members by sequencing selected BAC clones using Illumina and PacBio approaches.

**Results:**

BAC insert assemblies identified 15 predicted genes that are organized into three clusters. Two of the gene clusters have almost identical flanking regions, suggesting that they may be non-matching allelic clusters residing at the same genomic locus. GA STRs surround all genes and appear in large stretches at locations of putatively deleted genes. GAT STRs are positioned at the edges of segmental duplications that include a subset of the genes. The unique locations of the STRs suggest their involvement in gene deletions and segmental duplications. Genomic profiling of the *Sp185/333* gene diversity in 10 sea urchins shows that no gene repertoires are shared among individuals indicating a very high gene diversification rate for this family.

**Conclusions:**

The repetitive genomic structure of the *Sp185/333* family that includes STRs in strategic locations may serve as platform for a controlled mechanism which regulates the processes of gene recombination, gene conversion, duplication and deletion. The outcome is genomic instability and allelic mismatches, which may further drive the swift diversification of the *Sp185/333* gene family that may improve the immune fitness of the species.

**Electronic supplementary material:**

The online version of this article (doi:10.1186/s12864-016-3241-x) contains supplementary material, which is available to authorized users.

## Background

Most regions of eukaryote genomes are generally stable, show few changes between generations and over evolutionary time, and are maintained by accurate DNA repair mechanisms [1]. In contrast, some genomic regions are regarded as unstable (also called “fragile” [2]) and show more rapid changes particularly when they appear in association with tandem sequence repeats [3–6]. These regions show a higher frequency of breakage and are subject to increased errors and mutation rates during homologous repair processes (reviewed in [7]). Genomic regions that contain repeats can show swift changes in sequence, of which some are subject to selection, leading to rapid genome evolution. Clustered arrays of duplicated genes with similar sequence can be identified computationally as large tandem repeats, and relative to the practical problem of genome assembly, these regions are particularly difficult to assemble from short sequencing reads [3, 8]. This type of unstable genomic organization can be found in immune gene families in a wide range of organisms including human killer immunoglobulin-like receptor (*KIR*) gene family [9], genes encoding the fibrinogen related proteins (FREPs) in fresh water snails [10], allorecognition (*alr*) genes in a marine hydroid [11, 12], and disease resistance (*R*) genes in higher plants ([13, 14], reviewed in [15]). The beneficial outcome of genomic instability in regions that harbor immune gene clusters is the appearance of new genes within a family that increase its diversity (reviewed in [16]), which, when under positive selection from pathogen pressure, may result in improved immune function for detecting and responding to different pathogens or symbionts, and thereby improving the fitness and survival of the host.

**Fig. 1:**
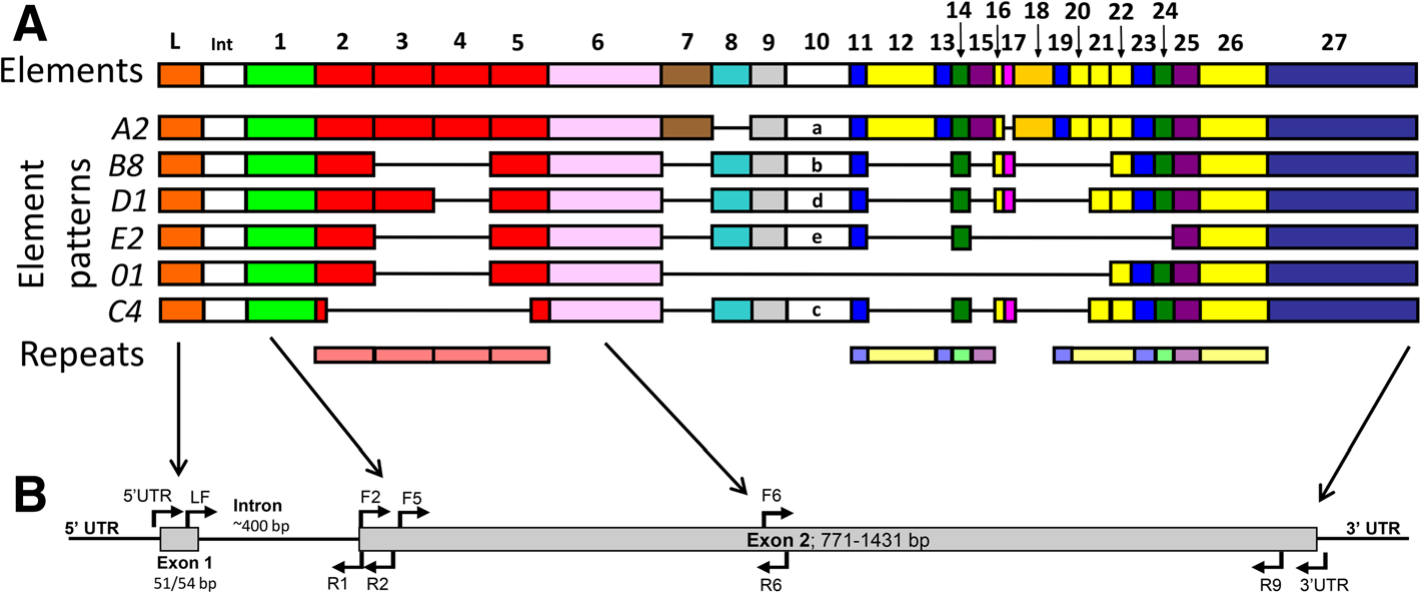
The *Sp185/333* genes have two exons and a mosaic of elements in the second exon. **a** An alignment cartoon illustrates the structure of several genes with two exons (shown in relative size scale) and one intron (int; not shown to scale). Elements in the second exon are indicated as colored rectangles and gaps that have been artificially inserted to optimize the alignment are shown as horizontal black lines. All known elements are numbered at the top. Element patterns share mosaics of elements and naming of element patterns (on the left) are based on the sequence of element 10 (equivalent of element 15 in [25]). The imperfect, tandem type I repeats in the 5′ half of the second exon are indicated as red rectangles (elements 2–5) and have been evaluated computationally for duplications, deletions and recombinations [31]. Five additional types of repeats are imperfect and interspersed in elements 11–26 (see [20]). This figure is modified from the repeat-based alignment published in [20]. **b** The approximate locations of primers are indicated with arrows within the standard *Sp185/333* gene structure. These primers are used to amplify *Sp185/333* gene sequences and to identify the genes within the BAC insert assemblies. Primer sequences are listed in Additional file 1: Table S1. The arrows between (**a**) and (**b**) indicate the correlation between elements in (**a**) and locations of primers shown in (**b**)

The purple sea urchin (*Strongylocentrotus purpuratus*) has a well characterized genome sequence, which is ~814 mb [17], was the first completed genome from a large, long-lived invertebrate, and has been updated three times to the current version (ver. 4.2; (http://www.echinobase.org)). This genome sequence is characterized by several large gene families that encode proteins with immune activities (e.g.*,* Toll-like receptors, NOD-like receptors, scavenger receptors, C-type lectins [18, 19]) and each of these families has undergone specific expansions within the echinoid lineage. Echinoid genomes also accommodate a unique immune response gene family, the *185/333* genes that have been partially characterized in two sea urchin species, *Strongylocentrotus purpuratus* (*Sp185/333*; [20, 21]) and *Heliocidaris erythrogramma* (*He185/333*; [22]). The *Sp185/333* genes function in the immune effector arm of sea urchin immunity and are strongly upregulated in response to different types of pathogens and PAMPs [23–28]. The *Sp185/333* genes have two exons, of which the first encodes the signal sequence, and the second of variable size among genes encodes the mature protein [20]. The second exon is composed of 25–27 blocks of sequences called elements (predicted from two equally optimal sequence alignments, see [20, 29]) that are present in different mosaic combinations resulting in 51 element patterns that have been identified to date (Fig. 1a) [20, 25]. Although the mechanism by which the mosaic element patterns are generated is unknown, this unique modular structure in the second exon results in great sequence diversity among the different genes and the encoded proteins [23, 27, 30], while maintaining a consistent general structure (Fig. 1b) [20, 29].

The size of the family is a point of considerable debate because gene prediction estimates based on the numbers of unique sequences isolated from individuals, the gene copy number in genomes obtained by qPCR, and estimates from the numbers of BAC clones isolated from two genomic libraries predict a family size of about 50 ± 10 [26, 29, 31, 32]. However, analyses of the *S. purpuratus* genome sequence (both the current and previous assemblies) indicate that the members of the *Sp185/333* family are largely absent with only five genes present [18]. Previous work has suggested that this discrepancy may be the consequence of the unique repetitive structure of the family and the sequence similarity among the genes that likely led to incorrect assembly of the gene family due to the collapse of multiple genes into a few loci of consensus sequence [21]. To obtain a more reliable evaluation of the *Sp185/333* gene family, Miller et al. [21] re-sequenced and re-assembled a single *Sp185/333*-positive BAC clone (GenBank accession number BK007096) and the verified insert sequence contained six *Sp185/333* genes clustered together within 34 kb. However, it was not known whether this BAC encompassed the entire family, whether additional clusters were present in the genome sequence, whether the clustered structure was typical of the entire family, whether all of the genes in the family were flanked by short tandem repeats (STRs), and whether additional pseudogenes (only one has been identified [20]) or gene fragments might be present within the assembled genome. To address this, a thorough screen of the *S. purpuratus* BAC library (large inserts of ~140 kb that was constructed from sperm DNA from a single sea urchin, which was the basis for the assembled genome sequence [33]) identified BACs with *Sp185/333* sequences. Selected BAC inserts were evaluated by next generation sequencing using both short read (Illumina) and long-read (Pacific BioSciences; PacBio) platforms. We report here that the *Sp185/333* family is present in an unusual repetitive genomic structure with three gene clusters that contain two to seven genes. Two types of STRs surround the genes, are present in locations of putatively deleted genes, and flank large segmental duplications. BAC insert sequences harboring two gene clusters show sequence matches in the flanking regions outside of the gene clusters suggesting that the sequences may reside within the same genomic locus and indicating a mismatch among alleles on homologous chromosomes. No pseudogenes or gene fragments are present in the genome sequence, which is unusual for tightly clustered genes with shared sequence. Evaluation of the *Sp185/333* gene content within genomes of 10 sea urchins using fragment length analysis shows exceptional diversity among individuals including unique gene sizes and repertoires. We propose that this genomic structure with shared sequences among tightly linked genes, STRs associated with gene duplications, deletions and segmental duplications and allelic regions with mismatched genes, is highly unusual, is likely a basis for very swift changes to the gene family that is selected over generations through interactions with pathogens (as has been identified in other systems (reviewed in [16]) and is likely to benefit the species by improving its immune fitness.

## Results

### *Sp185/333-*positive BAC clones were identified in the sea urchin large insert BAC library

The *Sp185/333* gene family in the *S. purpuratus* genome sequence (ver. 3.1; June 15^th^, 2011; (http://www.echinobase.org)) shows unexpectedly few members [21, 29]. This was likely due to the repetitive nature of the *Sp185/333* genes and flanking sequences [20, 21] that led to gene collapse by the assembly algorithm and the formation of artificially hybrid *Sp185/333* gene sequences. To address this problem, to identify as many of the members of the *Sp185/333* gene family as possible, and to characterize the structure of the family, a screen of the large insert (~140 kb) arrayed BAC library (25X genome coverage [33]) was conducted as described [20] and 75 *Sp185/333*-positive BAC clones were identified. To identify a subset of BAC clones for sequencing and insert assembly, clones were initially evaluated by PCR amplification using four primer sets (F2/R6, F6/R9, R2/R9, F5/R1; Fig. 1b; Additional file 1: Table S1) and the resulting amplicon sizes were used to verify the existence and sizes of *Sp185/333* genes, and to identify BAC clones that may have spanned the same genomic sequence (as expected based on library coverage). Of the 75 BAC clones that were evaluated, 27 supported PCR amplification and resulted in six different *Sp185/333* gene amplicon patterns (Additional file 2: Figure S1A). Patterns 1–3 were found in six, eight and nine BAC clones, respectively, whereas patterns 4–6 were only in one or two BACs each. In accordance with [21], PCR analysis of the intergenic distances in the BACs indicated that the majority of the genes were about 3.0–3.5 kb apart (Additional file 2: Figure S1B). The 48 BAC clones that did not support PCR for *Sp185/333* sequences may have lacked annealing sites for the primers, and consequently they were rescreened by Southern blot for *Sp185/333* sequences. Of these BAC clones, only BACs 3020I13 and 4069G2 showed visible bands for all restriction digests (*Not*1 plus either *Sal*I, *Xho*1 or *Bam*HI) and BAC 4069C2 showed a weak band only in *Sal*1/*Not*1 digest (Additional file 3: Figure S2). Variations in the positions of restriction sites suggested differences in the sequences among these BACs. These results indicated that the *Sp185/333* gene family in the sequenced genome of *S. purpuratus* was likely included in 30 BAC clones. The remaining 45 BACs were not evaluated further.

### Three *Sp185/333* gene clusters were identified in the *Sp185/333*-positive BAC clones

**Fig. 2:**
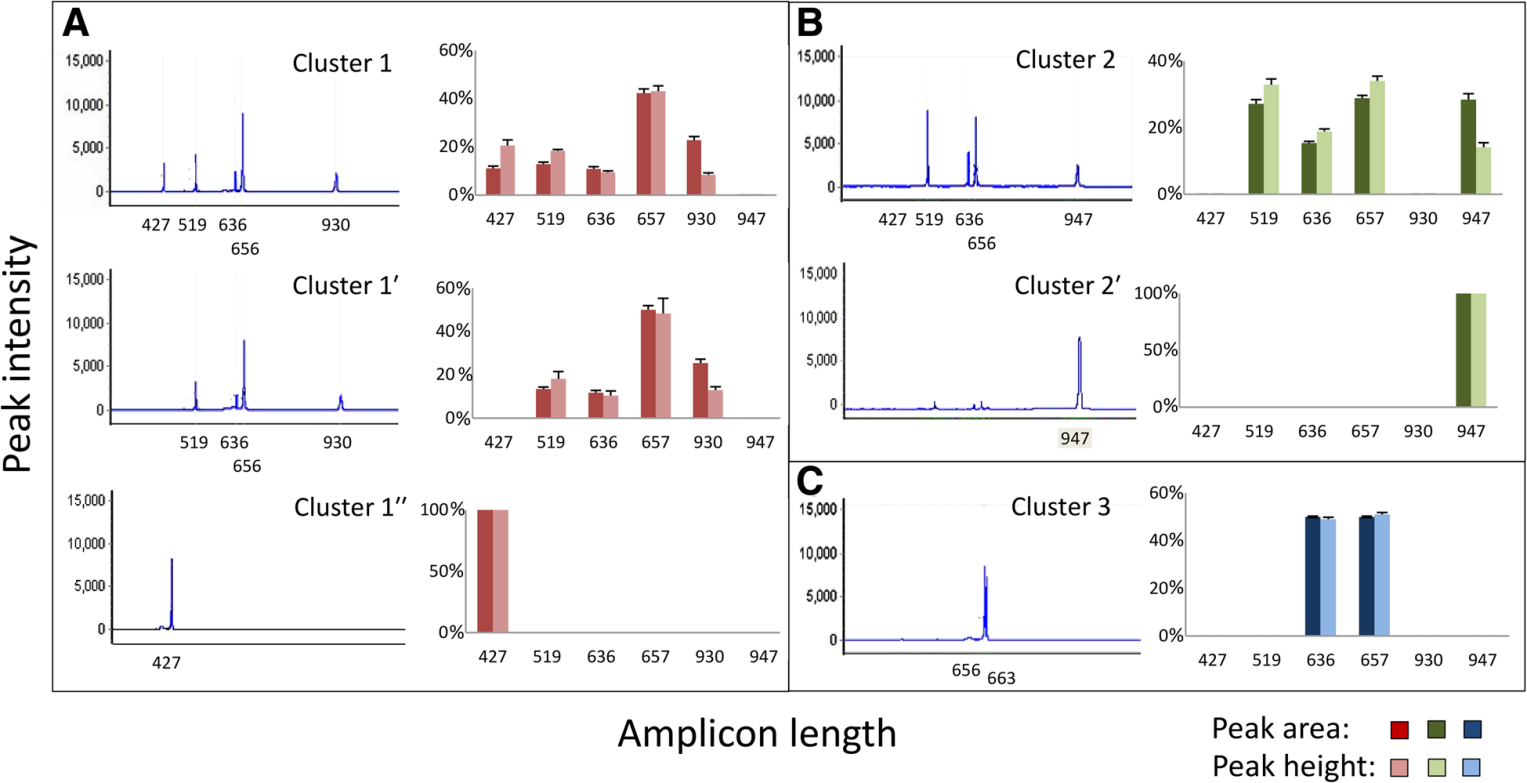
Three distinct *Sp185/333* gene amplicon size patterns are detected in large insert BAC clones. Amplicon peak height and area indicate three major amplicon size patterns from fragment analysis, which are designated as Cluster 1 (**a**), Cluster 2 (**b**) and Cluster 3 (**c**). Amplicon size patterns identified less often (1 or 2 clones of 27 analyzed; see Additional file 3: Figure S2A) and that partially match amplicons in either Cluster 1 or 2 are designated as Cluster 1′, Cluster 1″ (**a**) or Cluster 2′ (**b**). Fragment length analysis of each cluster was carried out for each of the corresponding BACs (listed below) using primers F6 and R9 (see Additional file 1: Table S1; Additional file 2: Figure S1B). Means and standard error were calculated when multiple BACs were used for analysis of Clusters 1 and 2. Cluster 1 BACs are 10A2, 10B1, 10C6, 4074 J14, 4079E24, 61 M13. Cluster 2 BACs are 4C3, 4007 J10, 4024 N22, 4029 F3, 4091I3, 10 K9, 64C18, 4011G4. Cluster 3 BACs are 10C18, 10H9, 10H10, 10 M18, 3090I9, 3104 N4, 3104P4, 4028 F7, 4067A10. Cluster 1′ BACs are 3033E12, 4093A10. Cluster 1″ BAC is 4093A10. The Cluster 2′ BAC is 409O3

The *Sp185/333* genes have a wide range of sizes [20], however differences in length among some genes may only be a few nucleotides (nt), which cannot be detected by standard gel electrophoresis of gene amplicons (see Additional file 2: Figure S1A). Furthermore, three duplicated copies of genes with the *D1* element pattern (Fig. 1a) in a single BAC insert has been reported (Additional file 4: Figure S3A) [21]. Because amplifying duplicated genes that result in a single amplicon size does not provide reliable estimates of gene copy numbers in BAC inserts, we implemented fragment length analysis that is regularly used to identify microsatellite length polymorphisms in populations [34]. This approach increased the resolution of the estimated gene sizes (a proxy for element pattern; see Fig. 1a) and copy numbers in each BAC. The most variable region in the *Sp185/333* genes, which is the 3′ half of the second exon (Fig. 1a) [20], was amplified from BAC DNA with F6 and R9-FAM primers (Additional file 1: Table S1; Additional file 2: Figure S1B) to generate amplicons within the size limits of the capillary sequencer. This approach was validated with BAC clone R3-3033E12, which had been previously sequenced, assembled and verified [21] and chromatograms of four amplicon sizes identified accurately each of the *Sp185/333* gene element patterns (*A2*, *B8*, *D1* and *E2*; Fig. 1a, Additional file 4: Figure S3). This approach also identified the three copies of the *D1* genes based on the amplicon peak height and area, which was approximately three times that relative to the other peaks for this BAC clone. Based on the verification of this approach, fragment length analysis was used to evaluate the 27 BACs that supported PCR amplification of *Sp185/333* genes. Gel images of non-fluorescent amplicons (Additional file 2: Figure S1A) and fragment length chromatograms of fluorescent amplicons for each BAC suggested six patterns of peaks with one to five amplicon sizes that were characteristic of different sets of BACs (Fig. 2). The signatures of the amplicon patterns for each BAC were characterized by both the amplicon length plus each fragment peak height and area, and the ratio of each peak relative to the tallest peak in the chromatogram for each BAC clone. Results were similar to the gel electrophoresis analysis showing three gene amplification signatures (Fig. 2) that were identified in 23 of 27 BACs and designated as Clusters 1, 2 and 3 (equivalent to patterns 1–3; Additional file 2: Figure S1A). In addition, three less common patterns were identified in only four of the BACs that partially overlapped either Cluster 1 or 2 and were designated as Clusters 1′, 1″ and 2′ (equivalent to patterns 4–6; Additional file 2: Figure S1A). Amplicon sizes reflect directly the element patterns of the genes and were used to predict different sets of genes. Results from this initial analysis of the *Sp185/333*-positive BACs suggested the *Sp185/*333 genes were arranged in at least three clusters.

### BAC inserts were assembled using two high through put sequencing approaches

#### Illumina sequencing fails to resolve the *Sp185/333* gene clusters

To assemble the sequence of the *Sp185/333* gene family, one approach was to greatly increase the sequence coverage to enable increased assembly stringency, which was permitted by Illumina sequencing. Eight BAC clones were selected for sequencing using the Illumina platform based on their predicted *Sp185/333* gene signatures from the fragment length analysis (Cluster 1, 10B1, 4074J14; Cluster 2, 10K9, 4024N22; Cluster 3, 10M18, 3104P4; Cluster 1″, 14K16; Cluster 2′, 4092O3). BAC clones corresponding with Cluster 1′ were omitted because this region corresponded to previously sequenced BAC R3-3033E12 (GenBank BK007096; Fig. 2) [21]. Three additional BAC clones (3020I13, 4069C2, 4069G2) were chosen based on results from Southern blots (Additional file 3: Figure S2B). All together, eleven BAC clones were sequenced with the Illumina platform.

Sequences for eleven BAC clones were assembled independently of each other (Table 1). Of these, only six assembled into a single contig of which some contained up to eight sequence gaps, whereas the remaining five BAC assemblies resulted in five to 24 unoriented contigs. Searches for the *Sp185/333* gene sequences and characterization of the structures of the clustered genes were carried out on the six assemblies with a single contig and covered the three *Sp185/333* gene clusters predicted by fragment length analysis (Fig. 2). Verification of these Illumina assemblies by searches for *Sp185/333* primer sequences identified significant assembly problems within the gene clusters including incomplete and fragmented *Sp185/333* genes. Four of the five assemblies (with the exception of BAC clone 3104P4) did not contain all of the expected primer sequences within the genes and did not match the expected gene sizes and numbers predicted by PCR and fragment analysis (Fig. 2). Furthermore, the BAC insert assemblies were shorter than expected compared to results from the pulsed field gel electrophoresis (PFGE) evaluation of insert sizes (data not shown). On the other hand, the assembly for BAC clone 3104P4 contained two complete genes of two sizes that were verified by the locations of the primer sequences, by PCR amplicon sizes and by fragment length analysis, which were in accordance with all predictions suggesting that this Illumina based assembly may have been correct.

**Table 1:** BAC insert assemblies based on Illumina and PacBio reads

Accession number	BAC clone	Cluster^a^	Sequencing technique^b^	No. of contigs	Length (nt)	Final length^c^	Gaps^d^	N50 (nt)	Comments
KU668451	10B1	1	Illumina	1	153857		8	153857	Both ends confirmed
			PacBio	1	166299	157472	0	166299	
KU668452	4074J14	1	Illumina	1	136199		8	136199	Both ends confirmed
			PacBio	1	151419	142396	0	151419	Artificial duplication at the ends
KU668453	10K9	2	Illumina	1	148572		2	148572	Both ends confirmed
			PacBio	1	153438	144627	0	153438	Artificial duplication at the ends
KU668450	10M18	3	Illumina	1	122168		8	122168	Both ends confirmed
			PacBio	1	136808	74402	0	136808	
KU668454	3104P4	3	Illumina	1	118285		7	118285	Both ends confirmed
			PacBio	1	127392	118584	0	127392	Artificial duplication at one end
Not submitted	14K16	1″	Illumina	5	99921		3	58593	Ends missing
Not submitted	3020I13	SB^+e^	Illumina	1	56106		0	56106	Finished, both ends confirmed
Not submitted	4024N22	2	Illumina	7	293347		31	77201	One end missing
Not submitted	4069G2	SB^+^	Illumina	10	125037		2	54882	Both ends confirmed
Not submitted	4069C2	SB^+^	Illumina	24	27398		0	1016	Very poor assembly, one end missing
Not submitted	4092O3	2′	Illumina	6	279956		7	125559	Both ends missing

^a^See Fig. 2

^b^BACs sequenced by both techniques were used to characterize the *Sp185/333* gene family structure

^c^Final length was determined after corrections to remove artificial repeats generated from the assembly process and vector contamination

^d^Gaps are defined as spaces in the assemblies with unknown nucleotides that are indicated in the sequence as Ns

^e^SB^+^; positive for *Sp185/333* sequences by Southern blot (see Additional file 3: Figure S2B)

#### PacBio sequencing resolves the *Sp185/333* gene cluster sequences

**Fig. 3:**
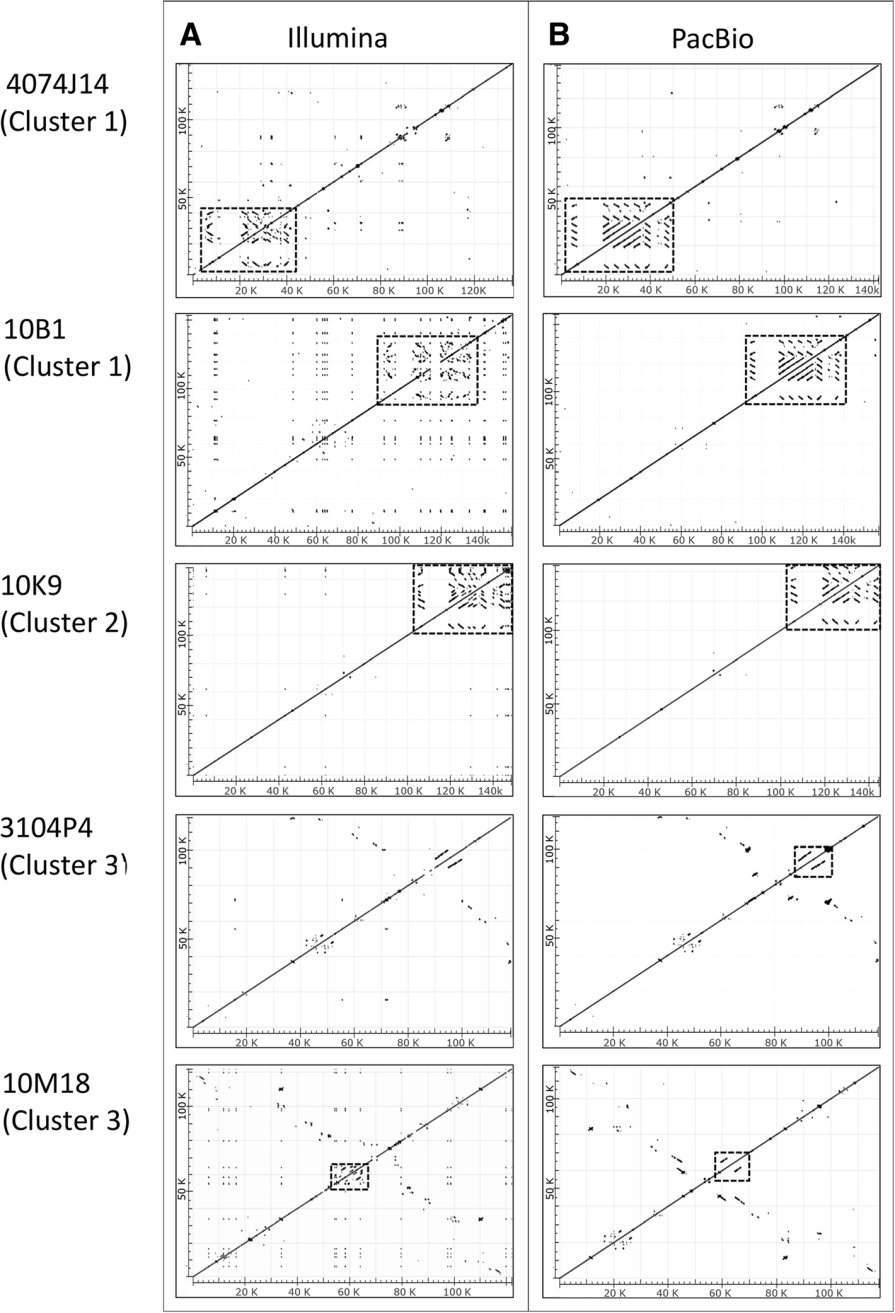
Repetitive sequences in the *Sp185/333* gene clusters are assembled correctly by PacBio but not by Illumina. Five BAC insert assemblies are compared to self by pairwise alignments and illustrated as dot plots. Lines and dots that are displaced from the central diagonal are repetitive sequences that include *Sp185/333* gene sequences (regions with displaced diagonals are indicated with boxes) and STRs (dots). **a** The *Sp185/333* gene clusters assembled from Illumina reads for BAC assemblies corresponding to Clusters 1, 2 and 3 have *Sp185/333* genes that are fragmented and shorter than expected (the displaced diagonals are composed of short lines or dots) based on gene size predictions (not shown) and according to previous reports [20, 21]. **b** The *Sp185/333* gene clusters assembled from PacBio reads for BACs compared to self. The PacBio assemblies show intact and longer displaced diagonals representing genes of expected length. The orientations of the genes are indicated by the displaced diagonals are either parallel (same orientation) or perpendicular (opposite orientation) to the central diagonal

Because the insert assemblies based on Illumina reads did not resolve the sequences of the *Sp185/333* gene clusters in 10 of 11 of the BAC insert assemblies, five BAC clones (10B1, 10M18, 3104P4, 4074J14, 10K9) were selected for re-sequencing by PacBio. BAC clones chosen for re-sequencing were based on whether the Illumina assemblies resulted in a single contig (Table 1) and if the gene clusters agreed with results from fragment length analysis (Fig. 2). Because PacBio sequence reads are significantly longer than both the Illumina reads and the sizes of the repeats associated with the *Sp185/333* genes, reads were expected to include non-repetitive sequence flanking the repetitive regions that could be used as anchors for improved assemblies. Each bar-coded PacBio library resulted in 3 to 17 × 10^3^ reads with an average read length of 6.4–7.5 kb. All five PacBio BAC assemblies resulted in a single contig without gaps (Table 1) and four of the five BACs (with the exception of 10M18) showed complete *Sp185/333* gene sequences of expected length based on the positions of *Sp185/333* primer sequences in the assemblies. The PCR amplicon sizes for genes and intergenic regions matched to the corresponding sequence sizes in these four assemblies and alignments showed expected full-length gene sequences separated by intergenic regions (represented as dot plots in Fig. 3). Results were supported by comparisons of BACs 10B1 and 4074J14 insert assemblies (not shown) to the overlapping and previously sequenced BAC insert (GenBank BK007096) [21].

### The BAC insert assemblies represent two loci in the genome

**Fig. 4:**
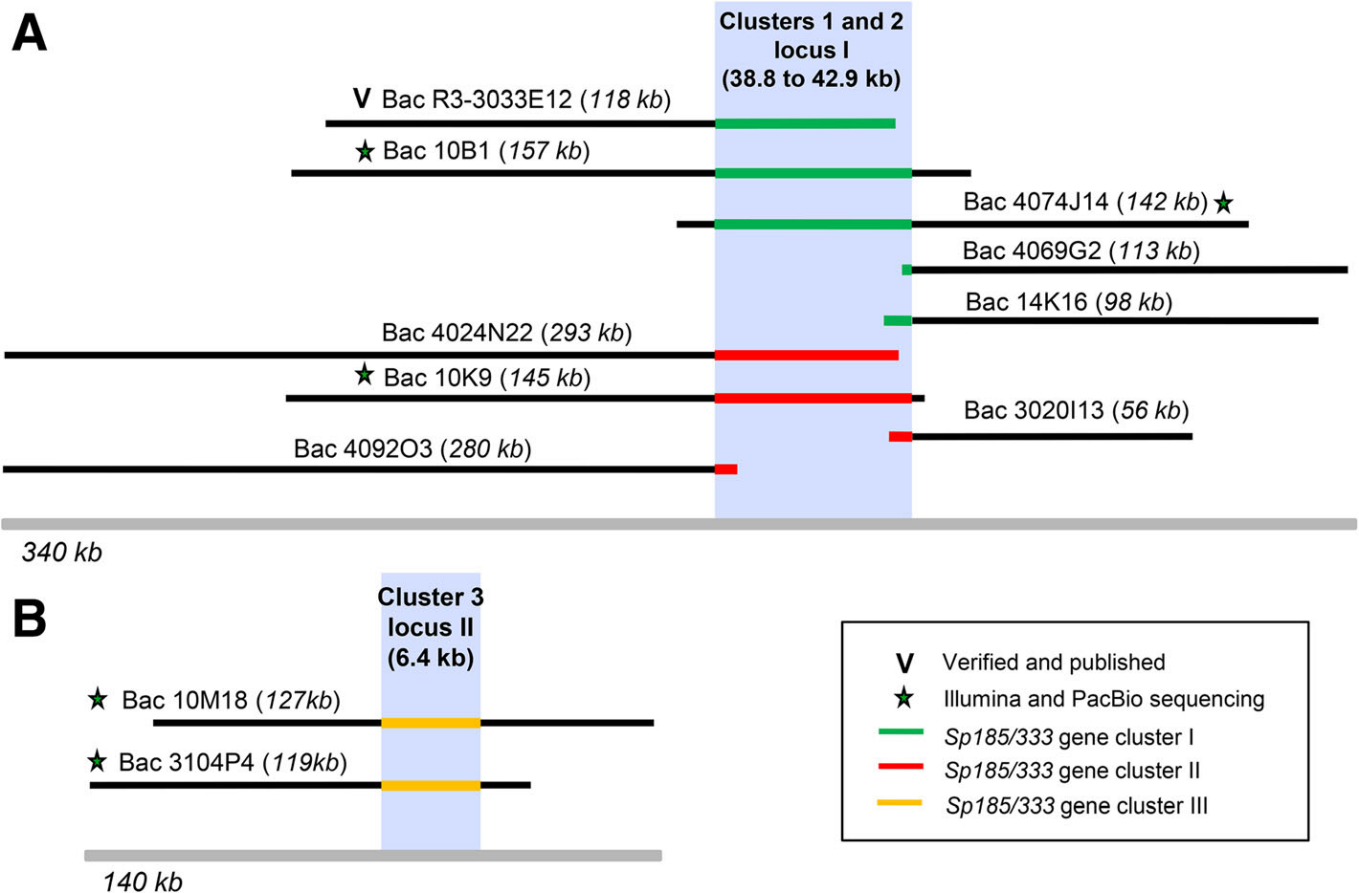
Comparisons among BAC insert assemblies suggest two genomic loci for three *Sp185/333* gene clusters. A map of all BAC insert sequences is based on pairwise alignments among all BAC insert sequences and indicates that Clusters 1 and 2 may be allelic. **a** Nine BAC insert sequences, including five that contain *Sp185/333* gene Cluster 1 and four that contain *Sp185/333* gene Cluster 2, match almost perfectly within the regions that flank the gene clusters. **b** The two BAC inserts that include Cluster 3 align separately as a different locus

To determine whether all of the BAC insert assemblies represented a single locus as inferred from the single cluster of *Sp185/333* genes in the genome sequence, or whether additional loci could be identified, all BAC sequences assembled from either PacBio and/or Illumina reads were aligned to each other. The regions flanking the gene clusters that did not contain repeats and that assembled more readily were used primarily to determine the positions of matches and overlaps among the sequences. Alignments of the flanking regions indicated either ~99 % sequence identity or no match (represented by dot plots, Additional file 5: Figure S4). When all BAC insert sequences were mapped relative to each other overlaps among the sequences were identified and indicated the presence of two separate loci (Fig. 4). Locus I encompassed nine BACs that either fully or partially overlapped and was composed of Clusters 1 and 2, including the *Sp185/333* gene cluster previously reported (R3-3033E12, GenBank accession number BK007096; [21]. The remaining two BACs overlapped to compose locus II, which covered Cluster 3 and was not present in the sea urchin genome sequence.

### The *Sp185/333* gene repertoire in the sea urchin genome sequence

**Fig. 5:**
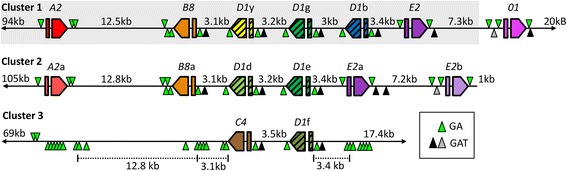
Three clusters of *Sp185/333* genes in the sequenced genome of *S. purpuratus*. The genes within Clusters 1, 2 and 3 range in size from 1170 to 1894 nt and are spaced apart by 3–12.8 kb. All genes have two exons, as indicated by the rectangle (first exon) and pentagon (second exon), which also indicates gene orientation within each cluster. Element patterns are listed above each gene and are indicated in different colors as initially defined by [21], which is identified by the shaded area in Cluster 1. All genes are surrounded by GA STRs (*green triangles*). The long stretches of GA STRs located on both sides of Cluster 3 are positioned at distances that correlate with corresponding genes in the other clusters. This map is based on three verified BAC assemblies (accession numbers: KU668452, KU668453, KU668454). Segmental duplications are surrounded by GAT STRs (*black triangles *denote GAT STRs of ≥ 35 repeats and *gray triangles *denote GAT STRs of 4–17 repeats; see Additional file 7: Table S3). Triangle location above or below the line indicates the orientation of the STRs, which is relative to the proximal gene

A detailed analysis of the BAC insert assemblies identified three clusters of *Sp185/333* genes (Fig. 5), which agreed with results from the fragment length analysis (Fig. 2). The clusters included 15 predicted genes (seven in Cluster 1, six in Cluster 2 and two in Cluster 3; Fig. 5) that ranged in size from 1170 to 1894 nt. The identification of two loci for the *Sp185/333* gene clusters based on matching flanking sequence for Clusters 1 and 2 (Fig. 4) strongly suggested that they may reside at the same locus in the genome and therefore may to be allelic. However, we could not rule out the possibility that Clusters 1 and 2 might be positioned within very large and recent duplications. Cluster 1 spanned 43 kb and contained seven genes with five different element patterns; *A2*, *B8,* three copies of *D1* (designated as *D1*y, *D1*g and *D1*b) and *E2* (see Fig. 1a for element patterns), of which all had been reported previously (indicated by gray shading in Fig. 5) [21]. An additional gene of the short *01* element pattern was located on an overlapping BAC and was ~400 nt beyond the right end of the previously reported BAC insert (see right – left orientation in Fig. 5). The five internal genes in Cluster 1 were tightly linked (3–3.4 kb apart), whereas the outer genes, *A2* and *01*, were separated from their nearest neighbor gene by ~12.5 and ~7.3 kb, respectively (Fig. 5). The orientation of the internal genes (*D1*y*, D1*g, *D1*b and *B8*) was in the opposite direction with respect to the flanking genes (*A2, E2* and *01*; Fig. 5). The allelic Cluster 2, which spanned 39 kb, had a similar structure, but with six genes of element patterns *A2* and *B8* (designated *A2*a and *B8*a), two copies of *D1* (designated *D1*d and *D1*e) and two copies of *E2* (designated *E2*a and *E2*b). The internal genes in Cluster 2 (*B8*a*, D1*d*, D1*e and *E2*a) were positioned 3.1–3.4 kb apart and the flanking genes, *A2*a and *E2*b, were separated from the nearest neighbor genes by 12.8 and 7.2 kb respectively (Fig. 5). The differences in gene composition between Clusters 1 and 2 included the number of *D1* and *E2* genes plus the presence of *01* in Cluster 1. Similarities included the *A2* and *B8* genes in addition to the order, orientation and spacing among the genes. It is noteworthy that if these two clusters represent allelic regions, as suggested from matching flanking regions, they do not have a matching number of genes, which confuses the concept of alleles. It is not clear which of the duplicated *D1* and *E2* genes should be considered alleles and may only be determined by more detailed alignments, percent identities and gene phylogenies (Barela Hudgell and Smith, unpublished). Consequently, because of the difficulties in knowing which of the genes constitute alleles, they will therefore all be called genes.

Cluster 3 was quite different from Clusters 1 and 2; it was much shorter, spanning 6.4 kb, and contained only two genes of element patterns *C4* and *D1*f (Fig. 1a) that were in the same orientation and separated by 3.5 kb (Fig. 5). An allelic region for Cluster 3 was not identified in this study, which may be based on the possible bias of the BAC library, or that the allelic region may be so similar that allelic BAC sequences may appear the same. Although the three gene clusters were different with regard to sizes and numbers of genes, they showed strikingly similar organization with regard to gene orientation, their order relative to element patterns, and their intergenic spacing.

### STRs flank the *Sp185/333* genes

Previous results showed the existence of tandem and interspersed repeats within the second exon of the *Sp185/333* genes [20] plus STRs of GA and GAT sequences on both sides of the genes [21]. To determine whether this unique structure was consistent for the entire gene family, the sequences of the three *Sp185/333* gene clusters on BAC clones 10B1, 10 K9 and 3104P4 were evaluated for tandem sequence repeats of increasing size from 2 to 342 nt per repeat, which were identified in all clusters. STRs flanked every *Sp185/333* gene, and the GA STRs with 12–132 repeats were located 303–790 nt from the start codon and 312–792 nt from the stop codon of each gene (Fig. 5, Additional file 6: Table S2). When the GA STRs were evaluated on a single DNA strand in the BAC sequences, reading from left to right (see orientation in Fig. 5) without regard to the *Sp185/333* gene orientation, GA STRs (triangles above; Fig. 5) flanked the peripheral genes and complementary CT STRs (triangles below) flanked the internal genes for both Clusters 1 and 2. The GA STRs flanked both genes in Cluster 3, and long stretches of GA STRs with 39–1048 repeats that were positioned to the left of *C4* (~3.1 and ~12.8 kb) and to the right of *D1*f (~3.4 kb; Fig. 5). These regions correlated with the relative locations of the *A2/A2*a, *B8/B8*a and *E2*a/*D1*b genes in Clusters 1 and 2 (Fig. 5, Additional file 6: Table S2). The large GA STRs also included some TC repeats plus up to 10 % interspersed repeats of TA, GG or AA (Additional file 6: Table S2). The large GA STRs located to the left of *C4* also included regions of TC repeats and multiple smaller regions of non-STR sequences (Additional file 6: Table S2). Although tandem repeats are common in the sea urchin genome (http://www.echinobase.org/Echinobase/repeats) the STR locations on both sides of each of the 15 predicted *Sp185/333* genes in the three clusters and surrounding segmental duplications were unique, had not been identified in similar orientations to genes in other families, and reflected an unusual but repeatedly identified pattern in this gene family.

**Fig. 6:**
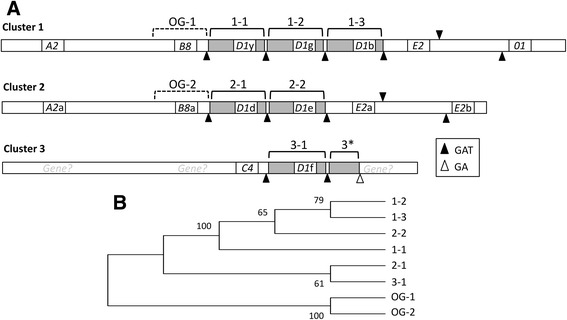
Tandem segmental duplications are bounded by GAT STRs. **a** Segmental duplications that include *D1* genes are present in all three clusters (*dark gray rectangles*), are bounded by GAT STRs (*black triangles* denote GAT STRs of 35 or more repeats; *gray triangles *denote 4–17 repeats), and are indicated by the Cluster number and order of appearance (1–1, 1–2, etc.). 3* indicates a partial segmental duplication in Cluster 3, which is flanked by a GAT STR on the left and GA STR on to the right. The locations of putatively deleted genes in Cluster 3 are marked as “*Gene?”* in light gray that correlate with the positions of long GA repeats (see Fig. 5). **b** An alignment of the full length segmental duplications was employed for phylogenetic analysis by maximum parsimony using MEGA. Bootstrap numbers are indicated and based on 500 iterations. The regions surrounding *B8* and *B8*a are defined as the outgroup (OG-1, OG-2). The sequences of the segmental duplications used for the alignment and phylogenetic tree are from BAC 10B1 (GenBank accession number KU668451) duplication 1–1 is nt 110752–115376; duplication 1–2 is nt 115460–120091; duplication 1–3 is nt 120177–124609; outgroup OG-1 is nt 106160–110660. BAC 10K9 (GenBank accession number KU668453) duplication 2–1 is nt 121690–126221; duplication 2–2 is nt 126365–130976; outgroup OG-2 is nt 117100–121600. BAC 3104P4 (GenBank accession number KU668454) duplication 3–1 is nt 91585–93433 and the partial duplication 3* is nt 96723–98626

Tandem segmental duplications bounded by GAT STRs that mostly included *D1* gene copies were another feature of the three *Sp185/333* gene clusters and were of similar size (4.2–4.6 kb; Fig. 6a). A partial segmental duplication of ~1.9 kb was present in Cluster 3 that did not include a gene (indicated as 3* in Cluster 3; Fig. 6a). All segmental duplications were positioned in the same orientation in each cluster and were located up to 100 nt apart. Phylogenetic analysis of the full length GAT bounded segmental duplications resulted in a clade composed of segmental duplications from Clusters 1 and 2 in a pectinate structure (see [35]) (Fig. 6b). The segmental duplication from Cluster 3 was most closely associated with one from Cluster 2 and both were positioned at the base of the tree in a sister clade. In general, the sequence similarities among the segmental duplications among the three gene clusters suggested that they may have originated from a single sequence.

In addition to segmental duplications that included the *D1* genes, GAT STRs (4–68 repeats) also surrounded the *E2* and *01* genes in Cluster 1 and the *E2*a gene in Cluster 2 (Fig. 5; Additional file 7: Table S3). The duplications that included *E2* and *E2*a were about the same length (~7.1 kb) and were highly similar (a pairwise distance of 0.005 based on an alignment using ClustalW in MEGA). A single flanking GAT STR (14 repeats) was located to the left of *E2*b in Cluster 2 (Figs. 5 and 6; Additional file 7: Table S3), however no flanking GAT STR was identified to the right of *E2*b based on searches of the overlapping BAC 3020I13 (Fig. 5). Consequently, the positioning of the GAT STRs surrounding *E2*b did not conform to the pattern identified for the *D1*, *E2* or *E2*a genes. The level of identity among the segmental duplications that included the *D1* genes and for the duplications harboring the *E2* and *E2*a genes suggested that the segmental duplications from all clusters may be recently generated.

### The *Sp185/333* gene family locus in the genome sequence is a hybrid of Clusters 1 and 2

The repetitive nature of the *Sp185/333* gene family created a significant genome assembly problem that caused the *Sp185/333* genes to be assembled artificially into five likely hybrid genes (genome assembly ver. 3.1, Scaffold 125; 02/18/2016) as predicted by [21]. Furthermore, allelic genes were purposefully omitted during genome assembly because they would have appeared as tandem gene duplications. Consequently, to determine which cluster was most similar to the *Sp185/333* gene cluster in the genome sequence, the five genes (*A1*, *B3*, *D1*, [*D1* or *D8*] and *E2*) in scaffold 125 (62.5 kb; 469500–531999 nt) plus the intergenic sequences and flanking regions were compared to the three clusters of genes reported here using YASS [36] (results not shown). The cluster in scaffold 125 had two *D1* genes, a single *E2* gene and did not include an *01* gene, and was therefore similar to both Clusters 1 and 2. The *A1* gene sequence in scaffold 125 was a hybrid of both *A2* in Cluster 1 and *A2*a in Cluster 2. Similarly, the *E2* gene sequence in scaffold 125 was a hybrid of *E2* in Cluster 1 and *E2*a in Cluster 2. The GA and GAT STRs were present in the expected positions in the gene cluster in scaffold 125, and the intergenic spacing was similar but not identical to the spacing in the BAC clusters. There were no similarities between Cluster 3 and the gene cluster in scaffold 125, and there was no evidence of Cluster 3 in the sequenced genome. In general, this comparison suggests that the *Sp185/333* gene cluster in scaffold 125 of the sequenced genome is likely a hybrid of both Clusters 1 and 2, which supports the possibility that these two clusters may be allelic.

### *Sp185/333* gene repertoire varies among individual sea urchins

The *Sp185/333* gene clusters reported here illustrated the unique DNA features of this family including gene clustering, shared sequences and unique positioning of STRs in agreement with [21] in addition to a possible allelic mismatch. These features are consistent with genomic instability [2, 3] that may drive *Sp185/333* sequence diversification among individuals. It is noteworthy that the *Sp185/333* genes that were randomly cloned and sequenced previously from three sea urchins demonstrated that different individuals may have different numbers and/or complements of *Sp185/333* genes as no identical gene sequences were shared among sea urchins [20, 29]. To investigate the possibility that the gene repertoire and copy number varied among individuals, fragment length analysis was employed to profile the *Sp185/333* gene family signature for genomic DNA isolated from sperm collected from 10 sea urchins. Results indicated that there were 14 amplicon sizes representing at least 14 different alleles of which nine amplicon sizes matched to genes with one or more element patterns, and five that did not match to sizes of any of the known 121 genes of unique sequence (Table 2) as reported by [20]. Five of the amplicon fragment sizes were shared among most of the animals and matched to gene sizes that have been commonly identified (*E*, *B, D*, C and *01* element patterns; Table 2). Of these common amplicon sizes, those matching to the *D1* element pattern size had the highest copy number in eight of 10 animals, which was consistent with *D1* genes being the most commonly identified gene from genomes of three sea urchins [20], in addition to the three clusters reported here and as reported previously [21]. A sixth common amplicon size of 940 nt was present in eight of 10 animals, but did not match to a known gene size. The other eight amplicon sizes were less common, only present in 1–4 animals, and matched to genes with *F1*, *A2* or *A2a* element patterns or were unknown (Table 2). The striking result from this analysis was that unique profiles of *Sp185/333* gene amplicon sizes and relative copy numbers were identified for each animal. Furthermore, because this technique only measures the size of a portion of the genes, these results were likely an under estimate of the sizes, diversity and repertoire of the genes (see footnotes to Table 2). The differences in the genomic signatures of the *Sp185/333* gene family among sea urchins suggested an unusually high genetic diversity in this family within individual sea urchins and inferred a very large repertoire of the *Sp185/333* genes among individuals in the population.

**Table 2:** *Sp185/333* allele distribution is unique for 10 purple sea urchins^a^

	Amplicon size^b^ and gene element pattern^c^													
Animal number	427 *01*	519 *E* ^d^	541 –^e^	617 *F1*	626 –^e^	636 *B* ^f^	643 –^e^	656 *D* ^g^	663 *C* ^h^	924 *A1*	928 –^e^	930 *A2*	940 –^e^	947 *A2a*
1	0.19	0.25				0.18		1.0	0.26			0.2	0.16	
2		0.77				0.22	0.18	1.0	0.87	0.32				
6		1.0				0.54		0.79	0.56	0.37			0.37	
7	0.27	0.59				0.33		0.87	1.0		0.43		0.4	
13	0.29	0.55				0.37		1.0	0.91			0.43		0.43
17	0.21	0.22	0.13			0.42		1.0	0.32		0.17		0.2	
18	0.58	0.55		0.37		0.69		1.0	0.33				0.78	
20	0.38	0.36				0.31	0.15	1.0	0.5	0.27			0.26	
25	0.12	0.3			0.12	0.1		1.0			0.12		0.12	
31	0.31	0.37				0.73		1.0	0.22		0.18		0.19	

^a^Distribution is expressed as peak area ratios relative to the largest peak in each animal. See Fig. 2 and Additional file 4: Figure S3

^b^Amplified with primers F6 and R9 (see Additional file 1: Table S1; Additional file 2: Figure S1B)

^c^Based on closest matches (≤3 nt difference) using 121 known unique *Sp185/333* gene sequences [20]

^d^Predicted gene element patterns include *E2*, *E3*, *E6*, *E8*, *E9* and *E10*

^e^No known gene element patterns match this amplicon size

^f^Predicted gene element patterns include *B2*, *B3* and *B8*

^g^Predicted gene element patterns include *D1, D5* and *D7*

^h^Predicted gene element patterns include *C2*, *C3* and *C6*

## Discussion

The *185/333* immune gene family in echinoids presents several unique but poorly understood genomic characteristics that are the focus of this study. The under representation of the *Sp185/333* gene family in the sequenced sea urchin genome is likely rooted in the fundamental technical problem that causes tandemly organized repetitive genomic sequences to be collapsed into artificial hybrid sequences or to be deleted from the assembly [3, 37]. Attempts to assemble the *Sp185/333* gene clusters using short reads generated by Illumina were generally poor and arose from repeats, a problem that was eliminated by employing long reads generating by PacBio. Similarly, when long reads were used to assemble a high quality genome for the western lowland gorilla, this approach solved the significant problem of incorporating repeats into the assembly and resulted in long N50 contigs [38]. Assemblies of echinoderm genomes could be improved significantly with long read sequencing, which is particularly evident from the poor assembly of the *Sp185/333* gene family in the current sea urchin genome sequence, which appears at a single locus in scaffold 125 as a hybrid sequence of Clusters 1 and 2, and does not include Cluster 3.

Based on the unusual characteristics of the clustered *Sp185/333* genes reported previously [21], we pursued additional sequences of the family to determine whether these characteristics were standard for this gene family. The general outcomes from long read sequencing technology following genomic library screening approaches verified several genomic features of the *Sp185/333* family that included gene structure and clustering, GA STRs surrounding all genes, and segmental duplications bounded by GAT STRs that are consistent with the previous study that was based on assembly from Sanger sequencing reads [21]. New genomic features of the *Sp185/333* gene family include the probable allelic mismatch between the *Sp185/333* genes in Clusters 1 and 2, the identification of Cluster 3, GA STRs positioned at putative gene deletion sites on both sides of Cluster 3, and GAT STRs associated with two types of segmental duplications that either carry *D1* or *E2* genes. The second exon of the *Sp185/333* genes is composed of a variety of mosaic combinations of elements (Fig. 1a), which imparts significant genetic variability within the *Sp185/333* gene family and is consistent with different *Sp185/333* genomic repertoires among individual purple sea urchins (Table 2) [20, 26, 29]. The expanded analysis of the *Sp185/333* gene family in 10 sea urchins provides evidence that the family is unstable, is highly variable in size and gene composition, and is unique among individuals. This is consistent with very preliminary results from Southern blots of genomic DNA from individual sea urchins probed for the *Sp185/333* sequences [26].

### *Sp185/333* family size prediction

The number of *Sp185/333* genes predicted for a given genome using three different estimates (reviewed in [29]) is 50 ± 10 genes, which is much greater than that reported here for the BAC assemblies and for scaffold 125 in the assembled sea urchin genome sequence. We screened the sea urchin large insert BAC library comprehensively for *Sp185/333*-positive clones employing several methods for BAC analysis. This effort resulted in three *Sp185/333* gene clusters that were repeatedly identified in a total of nine BACs that covered either all or some genes for each cluster. However, we cannot rule out the possibility that additional genes may have escaped identification, which is based on two additional genes isolated from the small insert BAC library that was constructed from the same genomic DNA as that used for the genome assembly [20]. It is feasible that both libraries had their own biases with regard to numbers of BACs with inserts harboring members of the *Sp185/333* gene family.

The low copy number of genes identified in the sea urchin genome sequence may also be an outcome of the assembly of the genome into scaffolds. The 42 chromosomes for *S. purpuratus* fall into three size ranges and are relatively similar within a given size range, but have not been characterized extensively as to the location of genes [39]. This type of gene mapping, which is typically done by in situ hybridization, is unavailable as a template onto which to map relative gene locations and linkage groups to assemble scaffolds into chromosomes. Furthermore, the assembly of a genome that is limited to the level of scaffolds can be the outcome of failures to assemble scaffolds across specific genomic regions that contain repeats [2, 3]. This may be attributed, in part, by additional *Sp185/333* gene clusters such as Cluster 3 that is not present in the genome sequence. Assembling a genome for an outbred, diploid organism is challenging and the outcome is likely to be a hybrid sequence of both haplotypes, as is the case for the hybrid gene sequences in scaffold 125 that are similar to both Clusters 1 and 2. Although the number of *Sp185/333* genes in the sequenced genome is lower than expected, it is possible that the individual sea urchin that was chosen for genome sequencing had a particularly small *Sp185/333* gene family. Variations in gene family size among individual sea urchins have been suggested previously [20, 29] as has been demonstrated here.

### STRs may drive duplications and deletions

The genes in the three *Sp185/333* clusters are organized in the same general order, orientation, and spacing, which leads to the notion that these clusters may have originated from a single ancestral gene or cluster that was significantly expanded by processes that include duplications and deletions of genes, segments and clusters into tandem or ectopic locations [7]. The proposed genomic instabilities may result, in part, from the presence of both GA and GAT STRs in the clusters. Historically, STRs have been considered “junk” DNA, however evidence suggests the functional importance of some non-coding DNA including promoting genomic instability and increased rates of changes to the DNA sequence [3–5]. The STRs themselves are prone to an estimated 10–100,000 fold higher mutation rates (that may include changes to nearby genes) compared to other regions of the genome that do not harbor STRs [4]. In the *Sp185/333* gene family, certain genes appear to have been duplicated more recently, including copies of both *D1* and *E2* genes based on both being positioned within almost identical segmental duplications. As has been suggested previously [21], the GAT STRs may be involved in the diversification process based on their unique positioning at the edges of the *D1* segmental duplications and some of the *E2* segmental duplications. Although the mechanisms by which this may occur is not understood, one possibility is that the duplications are the outcome of DNA breakage followed by repair that is based on misalignments to similar but non-identical sequences on the homologous chromosome [7]. During this process, chromosomal misalignment or meiotic mispairing, may lead to the deletion and duplication of genes by unequal crossing over between homologous chromosomes resulting in changes to the gene family size and composition in the subsequent generation. Segmental duplications of regions that include the *D1* genes in the BAC insert sequences are consistent with profiles of *Sp185/333* genes in all 10 sea urchins in which amplicon sizes that correspond to *D1* genes are present in a relatively higher copy number. On the other hand, the GA STRs that surround each of the *Sp185/333* genes may have important functions in genomic gene organization, including gene duplication and deletion [21]. Deletions are inferred from the long stretches of GA STRs that flank both sides of Cluster 3 and match precisely to locations of genes in Clusters 1 and 2. Furthermore, the combination of both segmental duplication and gene deletion may have been the basis for the partial segmental duplication in Cluster 3 (identified as 3*; Fig. 6a). This region matches the 5′ regions of the GAT STR segmental duplications that include *D1* genes but is missing the 3′ region of the segmental duplication that would be expected to include a gene. The missing 3′ region of this segmental duplication may have undergone a GA STR mediated deletion. However the underlying mechanisms for these processes are not understood.

The GA and GAT STRs are present in the clusters in both orientations, in accordance with the orientations of the genes, and constitute inverted repeats with complementary sequences. The formation of STR based secondary structures such as stem loops within the *Sp185/333* gene clusters may result in replication fork stalling during S phase of the cell cycle [5, 40], which can induce DNA double strand breaks [4]. This damage would be followed by repair with sequence from the homologous chromosome, resulting in sequence changes, mutations and gene recombination [4] among the *Sp185/333* gene clusters. Inverted complementary repeats may also be a basis, in part, for the genomic instability in the *Sp185/333* gene family, and examples of expected outcomes are hybrid *Sp185/333* genes resulting from recombination, which has been predicted from computational analysis of sequenced genes [31], and the mismatched alleles in clusters 1 and 2.

### There are no pseudogenes in the *Sp185/333* gene family

In addition to the STRs, the *Sp185/333* gene family has at least three other types of repeats: direct and interspersed repeats within the second exon, elements with shared sequences among genes, and segmental duplications. Similar to STRs, each one of these types of repeats may cause chromosomal misalignment during meiosis that may lead to variations in gene copy number plus sequence changes to individual genes resulting from homologous DNA repair processes. However, because these types of repeats appear within genes and include coding sequences (in contrast to the STRs), they may also lead to gene conversion, as has been suggested previously [7, 21]. The general lack of pseudogenes in the *Sp185/333* gene family (only a single pseudogene has been identified, although it was one of two intronless genes that may be retroposons [20]), is very unexpected for such tightly linked genes that share sequences and are surrounded by STRs (see [19]). One speculation is that gene conversion may correct pseudogenes in this family and that the STRs may block continued strand exchange and sequence homogenization of the entire cluster [21]. Because partial gene conversion may result in what might appear as gene recombination, potentially leading to frameshifts, missense sequence and early stop codons, the lack of pseudogenes in the *Sp185/333* gene family suggests that regulatory mechanisms may be involved.

### Immune gene diversification

All organisms must have means to stay ahead of and protect themselves against their pathogens and to control their commensals. This is particularly important given that microbes have multiple mechanisms for diversification [41], which are then selected for virulence based on improved ability to overcome immune responses and to promote colonization and infection. Typically, host protection in vertebrates is accomplished by diversifying the immune system, which includes the somatic recombination of the Ig gene family mediated by the recombination activating genes (*RAGs*) in jawed vertebrates [42] or the assembly of variable lymphocyte receptor genes in jawless vertebrates by activation induced cytidine deaminases (AID) [43, 44]. However, immune gene diversification also occurs in the absence of RAG and/or AID activity in many organisms and tends to be associated with clustered genes and genomic instability. This is evident from the *KIR* gene family, which is tightly clustered and shows variations in gene copy numbers for different human haplotypes in addition to sequence diversity in the population based on 15 to 112 alleles for different loci [9]. Variations in the *KIR* region is thought to result from genomic instability driven by shared sequences within and surrounding the genes, including minisatellite sequences in the first intron, all of which can lead to crossing over among the tandemly arranged genes, expansion and contraction of gene numbers from meiotic mispairing, in addition to recombination, gene conversion, domain shuffling, duplications and deletions and single nucleotide polymorphisms ([45, 46], reviewed in [47, 48]). Similar to the assembly problems for the *Sp185/333* gene family, the attributes of the *KIR* family have also resulted in very poor assembly in macaque genome sequences [49]. The major histocompatibility complex, with which the KIRs interact, is a cluster of duplicated genes that includes pseudogenes and is highly diversified in human populations with multitudes of polymorphic alleles, recombination hotspots and sequence variations [50]. In invertebrates, the colonial hydroid, *Hydractinia symbiolongicarpus* has two allorecognition (*alr*) loci, which control fusion and rejection among individuals, are positioned in genomic regions with pseudogenes and duplication that show sequence similarities to the expressed *alr* genes, in addition to putative transposable elements and regions of repetitive sequences [11, 12]. The Fusion/Histocompatibility (FuHC) locus in the colonial tunicate, *Botryllus schlosseri*, controls allogeneic fusion and rejection reactions among these sessile animals and shows sequence diversity, gene clustering, partial gene duplications and an extraordinary number of polymorphic alleles in the population for a subset of the genes (reviewed in [51]). Finally, excellent examples of repeat driven diversity are the anti-pathogen *R* gene families in higher plants, of which many are present in clusters of duplicated similar genes [15, 52]. *R* gene diversity results from unequal crossing over, gene conversion, duplications and deletions, all of which may lead to hybrid *R* genes or new genes with advantageous anti-pathogen functions [53]. The common theme among these examples is clustered similar genes (that may be considered as large repeats), plus repeats within and/or near genes, which together all lead to genomic instability. The emergence of new genes with new functions that are selected based on successful pathogen interactions and are therefore advantageous to the fitness and survival of the host reinforce and perpetuate the gene systems that function on the instability of specific genomic regions.

We have proposed here and in previous communications [7, 21, 31] a range of possible mechanisms that could result in sequence diversification of the *Sp185/333* genes in addition to variations in the size of the family among individuals. To date, diversification mechanisms that may result from regional instability in the genome have not been tested experimentally for the *Sp185/333* gene family (or in any other gene system that diversifies based on genomic instability) and our speculations are based on computational analyses of the sequences within and surrounding the genes. The rate of change within the *Sp185/333* genes can be deduced from the observations that there are no shared gene sequences among sea urchins, that there is a wide variety of mosaic element patterns, and that adjoining elements among genes may have different sequences [31]. Gene recombination can be inferred from the lack of correlation between the patterns of tandem repeats (elements 2–5, see Fig. 1a) compared to the patterns of interspersed repeats (elements 11–26, Fig. 1a) of the second exon among different genes. Detailed computational analyses of the *Sp185/333* gene sequences has inferred i) swift evolution of the tandem type 1 repeats located in the 5′ half of the second exon (Fig. 1a) based on predictions of duplications, deletions and recombinations, ii) rates of gene recombination that may be comparable to VJ recombination for the T cell receptor α locus, and iii) recombination that may occur at any point along the length of a gene [31]. In addition, there is no correlation among cDNA sequences when comparing the sequence of the first exon to the sequence of the adjacent 5′ untranslated region [24]. When the diversification rates of the *Sp185/333* genes are estimated based on molecular clock analysis, results suggest that the extant genes shared a common ancestor 2.7–10 million years ago, which overlaps with the estimate of *S. purpuratus* divergence from its sister species [29, 32]. This suggests ongoing recombination and that the extant genes are relatively young [31] in agreement with the *Sp185/333* gene family showing differences among different individuals. Although these computational predictions have not been tested experimentally, the results are consistent with observationsfor the gene family ﻿structure.

Clusters 1 and 2 appear to be allelic based on matching sequences that flank the clusters and the sequence of genomic scaffold 125 that is a hybrid of both clusters. However, the non-matching loci within the two clusters suggests larger scale genomic modifications that alter gene copy number and changes to the members of the clusters. Repetitive regions in genomes are associated with genomic instability, including meiotic mispairing that results in significant changes to gene clusters that share sequence and mispair during meiosis leading to unequal crossing over that alters the numbers of genes in one homologous chromosome relative to the other (reviewed in [7]). The outcome of such large scale changes can be observed as mismatched alleles and variations in the gene family size among individuals in the population. Many of these changes have been reported for *R* genes in higher plants [14, 15, 53], and the characteristics of many *R* gene families parallel those of the *Sp185/333* gene family [7]. When genomic changes resulting from meiotic mispairing, segmental duplications, gene duplications and deletions, gene conversion and single point mutations are considered together, the level and rate of sequence diversification for members of a gene family is likely to be astounding.

Clustered immune genes with shared sequences in a wide range of plants and animals show changes that can range in impact on the host from altered coding regions to the formation of hybrid genes and gene conversion. Although these changes lead to pseudogenes in most gene families, changes can also benefit the species when new gene sequences are selected as being advantageous based on non-self interactions that result in improvements to pathogen (or allogeneic) detection (e.g., see [54]). The genomic instability in the *Sp185/333* gene family and the apparent allelic mismatch between Clusters 1 and 2 may be the result of duplication/deletion events in the specific individual sea urchin that was employed for BAC library construction and genome sequencing. The outcome of such swift diversification of the *Sp185/333* gene family within the population can be deduced from the variety of gene amplicons derived from 10 different sea urchins in which only some fragment sizes are shared among individuals.

## Conclusions

The central differences in the genomic structure of the *Sp185/333* gene family compared to the examples of clustered immune response genes reviewed above is that the diversification rate appears to be particularly swift (see [29, 31]) and may be regulated given that pseudogenes are almost absent. This lack of pseudogenes is an unusual characteristic that is not understood, however the concept of sequence correction has been proposed as an outcome of active gene conversion that is limited by the positions of the flanking STRs [21]. We suspect that additional novel gene diversification processes may also be involved. Clues may be the unique positioning of the STRs [21] that flank genes and segmental duplications, and their locations at putative gene deletion sites. When the concepts of a dynamically changing *Sp185/333* gene family are combined with the notion that the encoded proteins may all have multitasking, anti-pathogen binding capabilities [28], the outcome is an innate immune system in the sea urchin that is highly sophisticated, complex and flexible. The dynamics of this gene family including rapid changes in its genomic organization and coding sequence may provide a significant evolutionary advantage to these invertebrates for their survival over millennia in their marine habitat.

## Methods

### BAC DNA isolation and *Sp185/333* gene verification

BACs (75) were identified by screening the large insert (~140 kb) sea urchin BAC library prepared from sperm DNA from a single animal [33] as reported in [21] and obtained from Eric Davidson and Andrew Cameron at the Center for Computational Regulatory Genomics, the California Institute of Technology. *Escherichia coli* (DH10B) transfected with BAC clones were cultured over night at 37 °C in 2xYT culture media [55] with 12.5 μg/ml chloramphenicol. BAC DNA was isolated by an alkaline lysis protocol as described [20], and the presence of the vector (pBACe3.6) was confirmed by PCR amplification using 3.6F and 3.6R primers (Additional file 1: Table S1). BAC inserts were released from pBACe3.6 vector by *Not*I (New England BioLabs) digestion, and evaluated for size by PFGE (1 % agarose, pulsed field certified, 6 V/cm; switch time of 1–15 s over 16 h) by comparison to the MidRange PFG Marker I (New England BioLabs). BAC DNA was subjected to multiple PCR amplification reactions using five sets of *Sp185/333* degenerate primer pairs including four sets to amplify the genes (5′UTR/3′UTR, F2/R6, F6/R9, F2/R9) and one pair to amplify the intergenic regions (F5/R1; Additional file 1: Table S1; Additional file 2: Figure S1B). Primers were designed previously based on 121 unique *Sp185/333* cloned gene sequences and had been tested for their ability to amplify different regions within and between the genes [20, 21]. PCR reactions of 20 μl and 27 cycles included 50–100 ng BAC DNA template, 1X GoTaq ready mix (Promega), 0.1 μM each primer using the appropriate annealing temperature for the primer pair (see Additional file 1: Table S1) with elongation time of 30 s to 4 min depending on the expected amplicon size.

### Southern blots

BAC DNA was double digested with *Not*1 plus either *Sal*I, *Xho*1 or *Bam*HI (New England BioLabs) and fragments were separated on a 0.7 % agarose gel. DNA was transferred to nylon membranes (GeneScreen Hybridization Transfer Membrane; PerkinElmer) by standard capillary blotting [55] and membranes were blocked with hybridization solution according to [56]. Blots were evaluated with ^32^P-riboprobes generated with either T3 or T7 RNA polymerase from three, linearized gene clone templates (2-034 (GenBank accession number EF607716), 4-1521 (EF607770) and 4-1543 (EF607784)) according to [20, 21]. The specificity of the ^32^P-riboprobe to hybridize with *Sp185/333* sequences was evaluated with three *Sp185/333* genes of different element patterns in addition to an *SpC3* cDNA [57] that served as the negative control (Additional file 3: Figure S2A). Probes were hybridized to the filters in hybridization solution at 42 °C with rocking overnight according to [56]. Membranes were washed in 4X, 2X and 1X SSC (20X SSC is 0.3 M Na citrate, 3 M NaCl, pH 7) with 1 % SDS at 65 °C, dried and exposed to X-OMAT AR film (Eastman Kodak) for 1 or 3 h at room temperature or 24 h at −70 °C with an intensifying screen.

### Genomic DNA isolation

Sperm was obtained from 10 sea urchins that were spawned﻿ by exposure to a non-lethal electric shock (6–10 volts). DNA was extracted from ~5 μl of dry sperm using DNeasy Blood & Tissue Kit (Qiagen) according the manufacturer’s instructions for blood extraction.

### Fragment length analysis

*Sp185/333* genes of different sizes and copy number in the BAC clones (50 ng) or from genomic DNA (5 ng) were estimated by fragment length analysis using F6 and R9 modified with 6-fluorescein amidite (R9-FAM; Integrated DNA Technologies; Additional file 1: Table S1; Additional file 2: Figure S1B) to amplify the 3′ half of the second exon of the *Sp185/333* genes. Labeled amplicons were analyzed by electrophoresis on a 1 % agarose gel to estimate quantities, and 0.5–2 μl of the product were mixed with 10 μl Hi-Di formamide containing 2.5 % GeneScan™ 1200 LIZ® size standard (ThermoFisher) and loaded onto an ABI PRISM 3130 Genetic Analyzer for capillary electrophoresis operated by ABI3130 Data Collection software (ThermoFisher). Peaks were analysed by GeneMarker® HID ver. 1.90 (SoftGenetics) based on length in nt, relative height and relative area of each peak according to the following calculation: peak intensity (height or area) divided by the tallest peak (or largest area) in a given sample, which resulted in fractional ratios of each individual peak in a sample.

### BAC sequencing and insert assembly

BAC DNA (20 μg) was isolated using the Large-Construct Kit (Qiagen). Contamination with *E. coli* (DH10B) genomic DNA was detected by amplification of two regions of the genome using primer pairs Ec1F/Ec1R and Ec2F/Ec2R (Additional file 1: Table S1). The level of contamination was determined by comparing *E. coli* amplicon intensity to amplicons of similar size from pBACe3.6 vector using primer pairs; 3.6-1F/3.6-1R and 3.6-2F/3.2-2R (Additional file 1: Table S1) that amplified two different genomic regions of 1 kb. *E. coli* DNA contamination that was 10 fold less than the amount of isolated BAC DNA was deemed sufficient for sequencing. Illumina sequencing and assembly for 11 BAC clones were conducted at the J. Craig Venter Institute (Rockville, MD). Two libraries were constructed for each BAC clone: a 500 nt paired-end library and a 5 kb mate pair library into which barcodes were incorporated to allow multiplexed sequencing. Subclone libraries from all BACs were sequenced on one lane of an Illumina MiSeq sequencer with an average read length of 250 nt. Reads were processed with BOWTIE2 [58] to remove matches to *E. coli* (DH10B) and pBACe3.6 sequences. Reads were separated by bar-code index, and adapter and primer sequences and poor quality regions (base quality < 10) were trimmed using TRIMMOMATIC [59]. Because read coverage varied greatly across different regions of individual BACs, coverage was normalized to 45X using DIGINORM (http://ged.msu.edu/papers/2012-diginorm), which had been used for the *Drosophila melanogaster* genome assembly [60]. Reads were assembled with ALLPATHS-LG (ver. R47449 [61]) with ploidy = 1 and MIN_CONTIG = 200. Intra-scaffold gaps were closed when possible using GapFiller [62]. The ligation sites between the pBACe3.6 vector and the insert were identified by reads that crossed these regions. The reads were assembled to reconstruct the vector ends; vecL was next to the *Eco*RI site at position 10 in the vector, and vecR was next to the *Eco*R1 site at position 2801 in the vector. Reconstruction of the vector – insert ligation sites allowed verification that the entire insert sequence had been obtained and enabled the orientation of the insert sequence relative to the vector.

Single molecule real time (SMRT; Pacific Biosciences, Menlo Park, CA) sequencing and assembly was carried out at the University of Maryland Genome Research Center (Baltimore, MD). DNA was sheared to 6–20 kb fragments using a g-Tube (Covaris) and libraries were constructed using the DNA Template Prep Kit and the DNA/Polymerase Binding Kit (Pacific Biosciences). Libraries were loaded onto SMRT Cells, and sequenced with the DNA Sequencing Kit (Pacific Biosciences). Assemblies of reads for each BAC were performed using Celera Assembler (ver. 82; (http://wgs-assembler.sourceforge.net/wiki/index.php?title=Main_Page)) and HGAP3 (SMRT Analysis ver. 2.3.0) using standard parameter sets. The read filtration procedure HGAP3 was used to assemble each bar-coded set of reads independently on each set of de-multiplexed reads. The best assembly was selected based on manual review and metrics including contig count, contig N50, assembly size compared to BAC digests evaluated by PFGE (see above), and gene profiles obtained by fragment length analysis (see above). Sequences that overlapped the insert and the vector were detected and Minimus2 [63] was used to overlap and trim the ends of the contigs. The final contig set for each BAC was improved using Quiver [64]. pBACe3.6 vector sequences at the edges of the insert were identified after assembly using VecScreen NCBI tool (http://www.ncbi.nlm.nih.gov/tools/vecscreen/) and removed manually. Correctness of the sequence outside the *Sp185/333* gene cluster was verified and corrected manually based on pairwise alignments for the SMRT and Illumina assemblies that covered the same region.

### Verification of BAC insert assemblies and identification of *Sp185/333* genes

The identification of *Sp185/333* gene sequences and their positions within the BAC insert assemblies was established using GenePalette [65] based on the presence and locations of the *Sp185/333* primer sequences (R1, F2, F5, F6, R6 and R9; Additional file 1: Table S1; Additional file 2: Figure S1B) within the two exons and the verification of the gene ends based on the conserved primer sequences at the 5′ and the 3′ ends of the genes (5′UTR, 3′UTR; Additional file 1: Table S1; Additional file 2: Figure S1B). Amplicon sizes (estimated by gel electrophoresis images and fragment length analysis) of intragenic and intergenic sequences were compared to sizes and distances predicted in the insert assemblies as calculated by the locations of the primer sequences. Pairwise alignments of the assemblies to self and to each other in addition to the generation of dot plots were done using Global align (https://blast.ncbi.nlm.nih.gov/Blast.cgi). The element pattern for each gene was identified from alignments to 121 known unique *Sp185/333* gene sequences [20] using BioEdit [66] and genes were named according to matches to previously characterized gene element patterns (see Fig. 1a) [26].

### Identification of segmental duplications and short tandem repeats

Segmental sequence duplications were identified by dot plot analyses of each assembly to itself using Global align (https://blast.ncbi.nlm.nih.gov/Blast.cgi). STRs were identified using Tandem Repeat Finder [67] with alignment parameters of match = 2, mismatch = 7, indels = 7, and a minimum alignment score = 40. The large STR regions located to the left and right of Cluster 3 were analyzed with the parameters of match = 2, mismatch = 3, indels = 5, and a minimum alignment score = 30. Searches for GATs used the parameters of match = 2, mismatch = 3, InDels = 5, minimum alignment score = 30, maximum period size = 3. STR sequences were screened by eye to identify internal repetitive sequences that did not conform to the major repeat sequence and that occupied up to 10 % of the STR sequence. Additional searches for GAT (three repeats) and GA (four repeats) STRs were done with GenePalette [65] including allowances for mismatches.

### Alignments and phylogenic trees

The segmental duplications bounded by GAT STRs and that included the *D1* genes were collected from the *Sp185/333* gene clusters and the regions surrounding *B8* and *B8a* of corresponding size and position as the *D1* segmental duplications were selected as outgroup sequences. Alignments were conducted using MEGA (ver. 7; (http://www.megasoftware.net)) using default parameters for ClustalW and were not improved manually. Phylogenic trees were generated by both maximum likelihood and maximum parsimony using pre-set parameters with 500 bootstrap iterations.

## Additional files

Additional file 1: Table S1. Primers. A list of primers used in this study. (DOCX 21 kb)
Additional file 2: Figure S1. Six patterns of amplicons are repeatedly identified from multiple *Sp185/333*-positive BAC clones. A. Intragenic amplification employed three different primer pairs (Additional file 1: Table S1; Additional file 2: Figure S1B). Three major amplification patterns (1–3) are shown for 6–9 BAC clones and three rare amplification patterns (4–6) are shown one or two BACs from a total of 27 BACs that supported PCR. B. Intergenic amplification patterns used a single pair of primers specific for *Sp185/333* sequences; F5 and R1 (Additional file 1: Table S1; Additional file 2: Figure S1B). The actual intergenic regions are shorter than the amplicons shown because the primers were positioned within the genes. The major amplicon sizes of 3.8–5.3 kb minus the reported gene size range of 1.2–1.9 kb [20] results in predicted minimum and maximum intergenic region sizes of 1.9–4.1 kb, which is within the expected range according to [21]. This panel is a composite of lanes from seven different gels to illustrate the amplicon sizes. M, the Hi/Low DNA standard is shown in kb. (DOCX 159 kb)
Additional file 3: Figure S2. Three BACs are positive for *Sp185/333* gene sequences by Southern blot. **A**. The ^32^P-RNA probe was generated according to [21] from linearized gene clones that were employed as templates (2-034 [GenBank acc. no. EF607716], 4-1521 [EF607770], 4-1543 [EF607784]; [20]). Target sequences on the blot are genes 2-034, 2-036 (EF607718) and 10-028 (EF607645) that were amplified by PCR from genomic DNA, inserted into the pCR4-TA vector [20], and released using *EcoR*1 restriction digests. The negative control was a cDNA clone in the pBluescript vector (pBsc-063X) that encoded a complement homologue, SpC3 [57], from which the insert was released with *Not*1/*Xho*1. Digests were loaded on the agarose gel in varying volumes as indicated, blotted and probed according to [26]. **B**. Restriction digests as indicated were performed for all *Sp185/333*-positive BAC clones that did not support PCR using primers specific for *Sp185/333* sequences. Digests were separated by gel electrophoresis and blotted. The ^32^P-RNA probe described in **A** hybridized to BAC clones 3020I13 and 4069G2 for all digests, whereas it hybridized to BAC clone 4069C2 only for the *Sal*1/*Not*1 digest. BAC clone numbers are indicated only for those BACs to which the probe hybridized. (DOCX 103 kb)
Additional file 4: Figure S3. Fragment length analysis is employed to estimate gene element patterns and copy numbers of *Sp185/333* genes. **A**. BAC R3-3033E12 (GenBank BK007096) has six *Sp185/333* genes with four different element patterns (*A2*, *B8*, *D1* and *E2*) including three copies of the *D1* genes [21]. **B**. The fragment length chromatogram shows four gene sizes based on amplicons of the 3′ variable end of the second exon (F6/R9-FAM primers; see Additional file 1: Table S1; Additional file 2: Figure S1B) as predicted from the BAC sequence. **C**. The heights of all peaks were compared to the highest peak for each sample and used to evaluate the gene copy numbers. The height ratio of peak 657 is approximately three times that of the other peaks predicting the presence of three copies of the *D1* genes, in agreement with the BAC insert sequence. Means and standard error were calculated based on repeating the fragment analysis on the BAC eight times. (DOCX 157 kb)
Additional file 5: Figure S4. The regions flanking Clusters 1 and 2 match almost identically in sequence. **A**. Comparisons among BAC insert assemblies harboring *Sp185/333* gene Clusters 1 and 2 show almost perfect matches in one flanking region of ~90 kb. The arrows indicate the matching flanking sequences outside of the non-matching *Sp185/333* gene Clusters 1 and 2. **B**. Comparisons between the BAC insert harboring Cluster 3 with inserts harboring Cluster 1 show only matches to the *Sp185/333* gene sequences. The regions of the gene clusters with shared sequence are outlined with dotted lines. (DOCX 192 kb)
Additional file 6: Table S2. GA/CT STRs in *Sp185/333* gene clusters. A list of dinucleotide STRs associated with the genes in Clusters 1, 2 and 3, including their sequence, distance from the gene, number of repeats, analysis scores based on the Tandem Repeat Finder [67], and the percentage of variations in the repeat sequence. (DOCX 29 kb)
Additional file 7: Table S3. GAT/CTA STRs in the *Sp185/333* clusters. A list of trinucleotide STRs associated with the genes in Clusters 1, 2 and 3 showing results as listed for Additional file 6: Table S2. (DOCX 19 kb)

## Abbreviations

alr: Allorecognition
BAC: Bacterial artificial chromosome
FREPs: Fibrinogen related proteins
FuHC: Fusion histocompatibility
kb: Kilo base
KIR: Killer immunoglobulin-like receptor
mb: Mega base
NCBI: National Center for Biotechnology Information
NK: Natural killer
NOD: Nucleotide oligomerization domain
nt: Nucleotides
PFGE: Pulsed field gel electrophoresis
qPCR: Quantitative polymerase chain reaction
*R*: Resistance genes
*SDS*: Sodium dodecyl sulfate
SMRT: Single molecule real time
SSC: Sodium chloride sodium citrate buffer
STR: Short tandem repeat
UTR: Untranslated region
V/cm: Volts per centimeter
