# Supplementary material for: Short tandem repeats, segmental duplications, gene deletion, and genomic instability in a rapidly diversified immune gene family
Source: BMC Genomics. 2016 Nov 9;17:900. doi: 10.1186/s12864-016-3241-x (PMC5103432; doi:10.1186/s12864-016-3241-x)
Supplement: Additional file 1: Table S1. — Primers. A list of primers used in this study. (DOCX 21 kb) [file 12864_2016_3241_MOESM1_ESM.docx]

**Additional file 1**

**Table S1: Primers**

| Primer pair | Target | Amplicon size (kb) | Sequence^4^ | Annealing temperature |
| --- | --- | --- | --- | --- |
| 3.6F  3.6R | pBACe3.6 | 0.1 | GCATGGCATACGGATTGG  GGTTTACGCAGTTTCGGC | 55^o^C |
| 3.6-1F  3.6-1R | pBACe3.6 | 1 | AGCCGTGTAACCGAGCATAGC  GGAACATGACGGTATCTGCGAG | 55^o^C |
| 3.6-2F  3.6-2R | pBACe3.6 | 1 | GGCTTGCATAACGCTGACCAC  GACTGGCCATTGAAACTGTTGC | 55^o^C |
| Ec1F  Ec1R | *E. coli* DH10B genomic DNA | 1 | CGAAGCGACTGGAGCATGTG  ACGCCACATTCGCCAATTC | 55^o^C |
| Ec2F  Ec2R | *E. coli* DH10B genomic DNA | 1 | GCATTGGTGTCGTAGTAGTAGGC  GCCACCGTTTCAACCAAG | 55^o^C |
| 5’UTR^[1](#_ENREF_1)^  3’UTR^[1](#_ENREF_1)^ | *Sp185/333* external primers | 1.2-1.9 | YTDTAGCATCGCAGAKACCT  WAATTCTACACCTCRGCGAC | 55^o^C |
| F2^[2](#_ENREF_2)^  R9^[2](#_ENREF_2)^ | 2^nd^ exon | 0.7 – 1.4 | AAGMGATTWCAATGAACKRCGAG  CTTHARGTGGTGAARATGTCG | 55^o^C |
| F2^[2](#_ENREF_2)^  R6^[2](#_ENREF_2)^ | 2^nd^ exon,  3′ half | 0.3 – 0.5 | AAGMGATTWCAATGAACKRCGAG  GCAGCATCAGTTTCTTCKTCTC | 58^o^C |
| F6^[2](#_ENREF_2)^  R9^[2](#_ENREF_2)^ or  R9-FAM^3^ | 2^nd^ exon,  5′ half | 0.4 - 1 | GAAGAAGAAACTGATGCTGCC  CTTHARGTGGTGAARATGTCG | 55^o^C |
| LF  R2 | *Sp185/333* intron | 0.4 | ATCRTYGCCATYSTGGCYG  CATTCCACCRGGCCTT | 56^o^C |
| F5^[2](#_ENREF_2)^  R1 | Intergenic region | 3.8 - 7.9 | GGAACYGARGAMGGATCTC  TCTSCATTCCAYCMGGCC | 56^o^C |

^1^For locations of *Sp185/333* primers in a standard gene, see Figure 1B. Unless otherwise stated, primers were purchased from Integrated DNA Technologies.

^2^Original description and use of these primers is in [21].

^3^F9 used in fragment length analysis was labeled with FAM.

^4^IUPAC nucleotide code is used here to designate degenerate positions.
