# Supplementary material for: Short tandem repeats, segmental duplications, gene deletion, and genomic instability in a rapidly diversified immune gene family
Source: BMC Genomics. 2016 Nov 9;17:900. doi: 10.1186/s12864-016-3241-x (PMC5103432; doi:10.1186/s12864-016-3241-x)
Supplement: Additional file 2: Figure S1. — Six patterns of amplicons are repeatedly identified from multiple Sp185/333-positive BAC clones. A. Intragenic amplification employed three different primer pairs (Additional file 1: Table S1; Additional file 2: Figure S1B). Three major amplification patterns (1–3) are shown for 6–9 BAC clones and three rare amplification patterns (4–6) are shown one or two BACs from a total of 27 BACs that supported PCR. B. Intergenic amplification patterns used a single pair of primers specific for Sp185/333 sequences; F5 and R1 (Additional file 1: Table S1; Additional file 2: Figure S1B). The actual intergenic regions are shorter than the amplicons shown because the primers were positioned within the genes. The major amplicon sizes of 3.8–5.3 kb minus the reported gene size range of 1.2–1.9 kb [20] results in predicted minimum and maximum intergenic region sizes of 1.9–4.1 kb, which is within the expected range according to [21]. This panel is a composite of lanes from seven different gels to illustrate the amplicon sizes. M, the Hi/Low DNA standard is shown in kb. (DOCX 159 kb) [file 12864_2016_3241_MOESM2_ESM.docx]

**Additional file 2: Figure S1: Six patterns of amplicons are repeatedly identified from multiple Sp185/333-positive BAC clones. A**. Intragenic amplification employed three different primer pairs (Additional Table 1; Figure 1B). Three major amplification patterns (1-3) are shown for six to nine BAC clones and three rare amplification patterns (4-6) are shown one or two BACs from a total of 27 BACs that supported PCR. **B**. Intergenic amplification patterns used a single pair of primers specific for *Sp185/333* sequences; F5 and R1 (Additional Table 1; Figure 1B). The intergenic regions are shorter than the amplicons shown because the primers were positioned within the genes. The major amplicon sizes of 3.8 to 5.3 kb minus the reported gene size range of 1.2 to 1.9 kb [21] results in predicted minimum and maximum intergenic region sizes of 1.9 to 4.1 kb, which is within the expected range according to [22]. This panel is a composite of lanes from seven different gels to illustrate the amplicon sizes. M, the Hi/Low DNA standard is shown in kb.
