# Supplementary material for: Short tandem repeats, segmental duplications, gene deletion, and genomic instability in a rapidly diversified immune gene family
Source: BMC Genomics. 2016 Nov 9;17:900. doi: 10.1186/s12864-016-3241-x (PMC5103432; doi:10.1186/s12864-016-3241-x)
Supplement: Additional file 3: Figure S2. — Three BACs are positive for Sp185/333 gene sequences by Southern blot. A. The 32P-RNA probe was generated according to [21] from linearized gene clones that were employed as templates (2-034 [GenBank acc. no. EF607716], 4-1521 [EF607770], 4-1543 [EF607784]; [20]). Target sequences on the blot are genes 2-034, 2-036 (EF607718) and 10-028 (EF607645) that were amplified by PCR from genomic DNA, inserted into the pCR4-TA vector [20], and released using EcoR1 restriction digests. The negative control was a cDNA clone in the pBluescript vector (pBsc-063X) that encoded a complement homologue, SpC3 [57], from which the insert was released with Not1/Xho1. Digests were loaded on the agarose gel in varying volumes as indicated, blotted and probed according to [26]. B. Restriction digests as indicated were performed for all Sp185/333-positive BAC clones that did not support PCR using primers specific for Sp185/333 sequences. Digests were separated by gel electrophoresis and blotted. The 32P-RNA probe described in A hybridized to BAC clones 3020I13 and 4069G2 for all digests, whereas it hybridized to BAC clone 4069C2 only for the Sal1/Not1 digest. BAC clone numbers are indicated only for those BACs to which the probe hybridized. (DOCX 103 kb) [file 12864_2016_3241_MOESM3_ESM.docx]

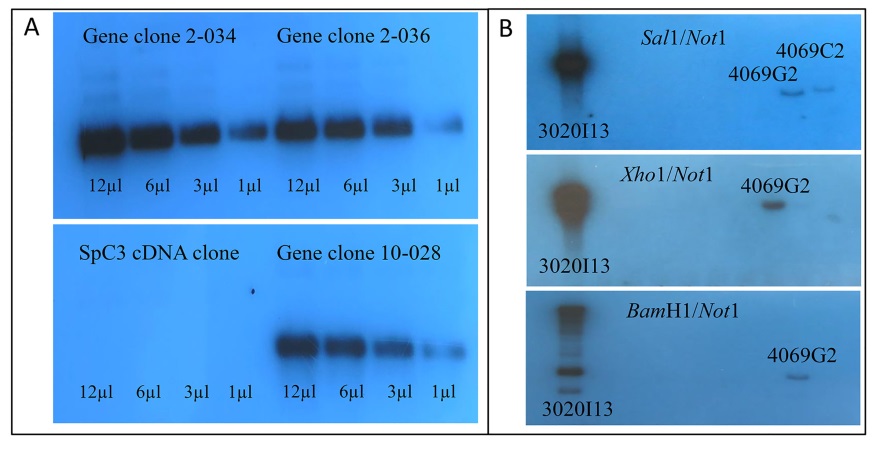


**Additional file 3: Figure S2: Three BACs are positive for *Sp185/333* gene sequences by Southern blot. A**. The ^32^P-RNA probe was generated according to [22] from linearized gene clones that were employed as templates (2-034 [GenBank acc. no. EF607716], 4-1521 [EF607770], 4-1543 [EF607784]; [21]). Target sequences on the blot are genes 2-034, 2-036 (EF607718) and 10-028 (EF607645) that were amplified by PCR from genomic DNA, inserted into the pCR4-TA vector [21], and released using *EcoR*1 restriction digests. The negative control was a cDNA clone in the pBluescript vector (pBsc-063X) that encoded a complement homologue, SpC3 [59], from which the insert was released with *Not*1/*Xho*1. Digests were loaded on the agarose gel in varying volumes as indicated, blotted and probed according to [27]. **B**. Restriction digests as indicated were performed for all *Sp185/333* BAC clones that did not support PCR using primers specific for *Sp185/333* sequences. Digests were separated by gel electrophoresis and blotted. The ^32^P-RNA probe described in **A** hybridized to BAC clones 3020I13 and 4069G2 for all digests, whereas it hybridized to BAC clone 4069C2 only for the *Sal*1/*Not*1 digest. BAC clone numbers are indicated only for those BACs to which the probe hybridized.
