# Supplementary material for: Short tandem repeats, segmental duplications, gene deletion, and genomic instability in a rapidly diversified immune gene family
Source: BMC Genomics. 2016 Nov 9;17:900. doi: 10.1186/s12864-016-3241-x (PMC5103432; doi:10.1186/s12864-016-3241-x)
Supplement: Additional file 4: Figure S3. — Fragment length analysis is employed to estimate gene element patterns and copy numbers of Sp185/333 genes. A. BAC R3-3033E12 (GenBank BK007096) has six Sp185/333 genes with four different element patterns (A2, B8, D1 and E2) including three copies of the D1 genes [21]. B. The fragment length chromatogram shows four gene sizes based on amplicons of the 3′ variable end of the second exon (F6/R9-FAM primers; see Additional file 1: Table S1; Additional file 2: Figure S1B) as predicted from the BAC sequence. C. The heights of all peaks were compared to the highest peak for each sample and used to evaluate the gene copy numbers. The height ratio of peak 657 is approximately three times that of the other peaks predicting the presence of three copies of the D1 genes, in agreement with the BAC insert sequence. Means and standard error were calculated based on repeating the fragment analysis on the BAC eight times. (DOCX 157 kb) [file 12864_2016_3241_MOESM4_ESM.docx]

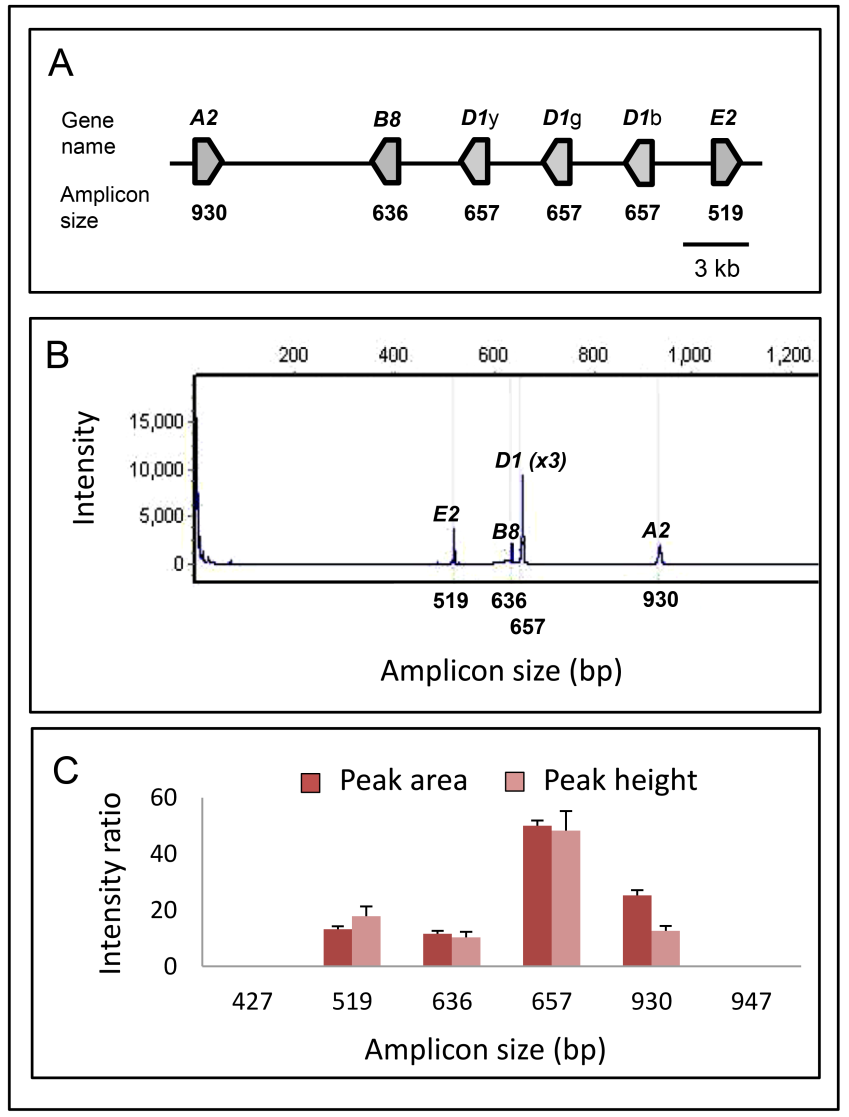


**Additional file 4: Figure S3: Fragment length analysis is employed to estimate gene element patterns and copy numbers of *Sp185/333* genes. A**. BAC R3-3033E12 (GenBank BK007096) has six *Sp185/333* genes with four different element patterns (*A2*, *B8*, *D1* and *E2*) including three copies of the *D1* genes [22]. **B**. The fragment length chromatogram shows four gene sizes based on amplicons of the 3′ variable end of the second exon (F6/R9-FAM primers; see Additional Table 1; Figure 1B) as predicted from the BAC sequence. **C**. The heights of all peaks were compared to the highest peak for each sample and used to evaluate the gene copy numbers. The height ratio of peak 657 is approximately three times that of the other peaks predicting the presence of three copies of the *D1* genes and is in agreement with the BAC insert sequence. Means and standard error were calculated based on repeating the fragment analysis on the BAC eight times.
