# Supplementary material for: Short tandem repeats, segmental duplications, gene deletion, and genomic instability in a rapidly diversified immune gene family
Source: BMC Genomics. 2016 Nov 9;17:900. doi: 10.1186/s12864-016-3241-x (PMC5103432; doi:10.1186/s12864-016-3241-x)
Supplement: Additional file 5: Figure S4. — The regions flanking Clusters 1 and 2 match almost identically in sequence. A. Comparisons among BAC insert assemblies harboring Sp185/333 gene Clusters 1 and 2 show almost perfect matches in one flanking region of ~90 kb. The arrows indicate the matching flanking sequences outside of the non-matching Sp185/333 gene Clusters 1 and 2. B. Comparisons between the BAC insert harboring Cluster 3 with inserts harboring Cluster 1 show only matches to the Sp185/333 gene sequences. The regions of the gene clusters with shared sequence are outlined with dotted lines. (DOCX 192 kb) [file 12864_2016_3241_MOESM5_ESM.docx]

**Additional file 5: Figure S4: The regions flanking Clusters 1 and 2 match almost identically in sequence.** **A**. Comparisons among BAC insert assemblies harboring *Sp185/333* gene Clusters 1 and 2 show almost perfect matches in one flanking region of ~90 kb. The arrows indicate the matching flanking sequences outside of the non-matching *Sp185/333* gene clusters 1 and 2. **B.** Comparisons between the BAC insert harboring Cluster 3 with inserts harboring Cluster 1 show only matches to the *Sp185/333* gene sequences. The regions of the gene clusters with shared sequence are outlined with dotted lines.
