# Supplementary material for: Short tandem repeats, segmental duplications, gene deletion, and genomic instability in a rapidly diversified immune gene family
Source: BMC Genomics. 2016 Nov 9;17:900. doi: 10.1186/s12864-016-3241-x (PMC5103432; doi:10.1186/s12864-016-3241-x)
Supplement: Additional file 6: Table S2. — GA/CT STRs in Sp185/333 gene clusters. A list of dinucleotide STRs associated with the genes in Clusters 1, 2 and 3, including their sequence, distance from the gene, number of repeats, analysis scores based on the Tandem Repeat Finder [67], and the percentage of variations in the repeat sequence. (DOCX 29 kb) [file 12864_2016_3241_MOESM6_ESM.docx]

**Additional file 6: Table S2: GA/CT STRs in *Sp185/333* gene clusters**^1^

| *Sp185/333* gene | Gene orientation^2^ | Distance from the gene (nt) | Orientation to the gene | Repeat sequence^3^ | No. of repeats | Score^4^ | Repeat variations |
| --- | --- | --- | --- | --- | --- | --- | --- |
| **Cluster 1** | | | | | | | |
| *A2* | F | 441 | 5′ | GA | 22.5 | 45 |  |
| *A2* | F | 728 | 3′ | GA | 18 | 45 |  |
|  |  | 792 |  | GA | 131.5 | 119 |  |
| *B8* | R | 670 | 3′ | GA | 19 | 49 |  |
|  |  | 546 |  | GA | 43 | 41 | 7% GG |
|  |  | 320 |  | GA | 76 | 157 |  |
| *B8* | R | 393 | 5′ | GA | 31 | 49 |  |
| *D1*y | R | 541 | 3′ | GA | 27 | 45 |  |
|  |  | 347 |  | GA | 27 | 81 |  |
| *D1*y | R | 448 | 5′ | GA | 33 | 87 |  |
| *D1*g | R | 555 | 3′ | GA | 26 | 41 |  |
|  |  | 355 |  | GA | 30 | 93 |  |
| *D1*g | R | 447 | 5′ | GA | 14 | 68 |  |
| *D1*b | R | 551 | 3′ | GA | 26 | 41 |  |
|  |  | 355 |  | GA | 28 | 85 |  |
| *D1*b | R | 448 | 5′ | GA | 14 | 38 |  |
| *E2* | F | 400 | 5′ | GA | 26.5 | 97 |  |
| *E2* | F | 406 | 3′ | GA | 18.5 | 40 |  |
| *01* | F | 1836 | 5′ | GA | 24 | 37 |  |
|  |  | 371 |  | GA | 28 | 85 |  |
| *01* | F | 325 | 3′ | GA | 15 | 62 |  |
|  |  | 493 |  | GA | 27 | 72 |  |
| **Cluster 2** | | | | | | | |
| *A2*a | F | 766 | 5′ | GA | 24.5 | 80 |  |
| *A2*a | F | 382 | 3′ | GA | 168.5 | 222 |  |
|  |  | 4834 |  | CT | 13 | 52 |  |
| *B8*b | R | 884 | 3′ | CT | 40.5 | 81 |  |
|  |  | 685 |  | GA | 14 | 47 |  |
|  |  | 645 |  | CT | 20.5 | 55 |  |
|  |  | 544 |  | GA | 16 | 64 |  |
|  |  | 334 |  | GA | 73.5 | 154 |  |
| *B8*b | R | 399 | 5′ | GA | 12 | 48 |  |
| *D1*d | R | 564 | 3′ | GA | 28 | 49 | 9% TG |
|  |  | 383 |  | GA | 21 | 66 |  |
| *D1*d | R | 408 | 5′ | GA | 19.5 | 60 |  |
| *D1*e | R | 563 | 3′ | GA | 26 | 41 | 10% TG |
|  |  | 367 |  | GA | 28 | 85 |  |
| *D1*e | R | 448 | 5′ | GA | 22 | 43 |  |
| *E2*a | F | 402 | 5′ | GA | 20.5 | 73 |  |
| *E2*a | F | 183 | 3′ | CT | 18.5 | 40 |  |
| *E2*b | F | 1753 | 5′ | GA | 25 | 41 |  |
|  |  | 371 |  | GA | 28 | 85 |  |
| *E2*b | F | 325 | 3′ | GA | 14.5 | 58 |  |
|  |  | 491 |  | GA | 35 | 113 |  |

| **Cluster 3** | | | | | | | |
| --- | --- | --- | --- | --- | --- | --- | --- |
| Left of Cluster 3 | R | 19838 |  | GA | 119 | 72 | 3% GG |
|  | F | 17356 |  | GA | 1047.5 | 1045 | 8% TA |
|  | F | 15655 |  | GA | 386 | 508 | 7% TA |
|  | F | 14891 |  | GA | 39 | 81 | 8% AA |
|  | F | 3973 |  | GA | 65 | 131 | 5% GG |
|  | F | 1762 |  | GA | 683 | 856 | 7% TA |
| *C4* | R | 322 | 3′ | GA | 62.5 | 164 |  |
| *C4* | R | 403 | 5′ | GA | 46 | 96 | 4% CA |
| *D1*f | R | 623 | 3′ | GA | 34.5 | 75 |  |
|  |  | 540 |  | GA | 17 | 50 |  |
|  |  | 365 |  | GA | 17.5 | 61 |  |
| *D1*f | R | 428 | 5′ | GA | 30.5 | 104 |  |
| Right of Cluster 3 | F | 2917 |  | GA | 395.5 | 349 | 7% TA |
|  | F | 4127 |  | GA | 690 | 529 | 7% TA |

^1^STRs were analyzed with Tandem Repeat Finder [73]. Parameters used were {(match = 2, mismatch = 7, InDels = 7), minimum alignment score =40, maximum period size = 2}.

^2^Gene orientation is based on Figure 5 in the main paper.

^3^All of the repeats are presented according to the orientation of the nearest *Sp185/333* gene.

^4^Alignment scores are calculated with the Tandem Repeat Finder.
