# Supplementary material for: Short tandem repeats, segmental duplications, gene deletion, and genomic instability in a rapidly diversified immune gene family
Source: BMC Genomics. 2016 Nov 9;17:900. doi: 10.1186/s12864-016-3241-x (PMC5103432; doi:10.1186/s12864-016-3241-x)
Supplement: Additional file 7: Table S3. — GAT/CTA STRs in the Sp185/333 clusters. A list of trinucleotide STRs associated with the genes in Clusters 1, 2 and 3 showing results as listed for Additional file 6: Table S2. (DOCX 19 kb) [file 12864_2016_3241_MOESM7_ESM.docx]

**Additional file 7: Table S3: GAT/CTA STRs in the *Sp185/333* clusters**^1^

| *Sp185/333* gene | Gene orientation^2^ | Distance from the gene (nt) | Orientation to the gene | Repeat sequence^3^ | No. of repeats | Score^4^ | | |
| --- | --- | --- | --- | --- | --- | --- | --- | --- |
| **Cluster 1** | | | | | | | | |
| *B8* | R | 488 | 5′ | GAT | 46 | 126 | | |
| *D1*y | R | 544 | 5′ | GAT | 65 | 193 | | |
| *D1*g | R | 529 | 5′ | GAT | 65 | 193 | | |
| *D1*b | R | 516 | 5′ | GAT | 35.7 | 60 | | |
| *E2*a | F | 590 | 3′ | CTA | 47.3 | 126 | | |
| *01* | F | 549 | 5′ | GAT | 16 | 46 | | |
| **Cluster 2** | | | | | | | | |
| *B8* | R | 544 | 5′ | GAT | 51 | | | 151 |
| *D1*d | R | 634 | 5′ | GAT | 36.6 | | | 74 |
| *D1*e | R | 513 | 5′ | GAT | 35.7 | | | 60 |
| *E2*b | F | 566 | 3′ | CTA | 48.3 | | | 132 |
| *E2*b | F | 517 | 5′ | GAT | 14.7 | | | 43 |
| **Cluster 3** | | | | | | | | |
| *C6* | R | 542 | 5′ | GAT | 51 | | 156 | |
| *D1*f | R | 529 | 5′ | GAT | 68 | | 201 | |

^1^STRs were analyzed with Tandem Repeat Finder [73]. Parameter used were {(match = 2, mismatch = 3, InDels = 5), minimum alignment score = 30, maximum period size = 3}.

^2^The gene orientation is based on Figure 5 in the main paper.

^3^All of the repeats are presented according to the orientation of the most proximal *Sp185/333* gene.

^4^Alignment scores are calculated according to Tandem Repeat Finder.
